# Supplementary material for: New Cadinane Sesquiterpenes from the Stems of Kadsura heteroclita
Source: Molecules. 2019 Apr 28;24(9):1664. doi: 10.3390/molecules24091664 (PMC6539153; doi:10.3390/molecules24091664)
Supplement: Supplementary file 1 [file molecules-24-01664-s001.pdf]

Article

New Cadinane Sesquiterpenes from the Stems of *Kadsura heteroclita*

Liang Cao[1,†], Nuzhat Shehla[1,2,†], Shumaila Tasneem[1,2], Mengru Cao[1], Wenbing Sheng[1], Yuqing Jian[1], Bin Li[1], Caiyun Peng[1], M. Iqbal Choudhary[2,*], Atta-ur-Rahman[2], Duan-fang Liao[1] and Wei Wang[1,2,*]

1 TCM and Ethnomedicine Innovation & Development International Laboratory, Innovative Materia Medica Research Institute, School of Pharmacy, Hunan University of Chinese Medicine, Changsha 410208, PR China; caoliang520945@126.com (L.C.); shehla_chm@hotmail.com (N.S.); tasneemshum@gmail.com (S.T.); caomengru0913@126.com (M.C.); wbs626@126.com (W.S.); cpujyq2010@163.com (Y.J.); libin_hucm@hotmail.com (B.L.); paudy@126.com (C.P.); dfliao@hnucm.edu.cn (D.L.)

2 H.E.J. Research Institute of Chemistry, International Center for Chemical and Biological Sciences, University of Karachi, Karachi-75270, Pakistan; aurahman786@gmail.com (A.U.R.)

* Correspondence: wangwei402@hotmail.com. (W.W.), Tel.: +86-136-5743-8606; iqbal.choudhary@iccs.edu. (M.I.C.), Tel: +92-21-4824924, 4819010

† These authors contributed equally to this work.

List of Tables and Figures

Table S1 ^{1}H and ^{13}C NMR spectroscopic data of compound (**4-7**)........ 4

Table S2. ^{1}H and ^{13}C NMR spectroscopic data of cytochalasin H (**8**)........ 5

Figure S1. ^{1}H NMR spectrum of compound **1** (CDCl3)........ 6

Figure S2. ^{13}C NMR spectrum of compound **1** ($CDCl_3$)........ 7

Figure S3. DEPT 90 ^{13}C NMR spectrum of compound **1** ($CDCl_3$)........ 8

Figure S4. DEPT 135 ^{13}C NMR spectrum of compound **1** ($CDCl_3$)........ 9

Figure S5. HSQC NMR spectrum of compound **1** ($CDCl_3$)........ 10

Figure S6. ^{1}H-^{1}H COSY NMR spectrum of compound **1** ($CDCl_3$)........ 11

Figure S7. HMBC NMR spectrum of compound **1** ($CDCl_3$)........ 12

Figure S8. NOESY NMR spectrum of compound **1** ($CDCl_3$)........ 13

Figure S9. HRESIMS spectrum of compound **1**........ 14

Figure S10. UV spectrum of compound **1**........ 15

Figure S11. IR spectrum of compound **1**........ 16

Figure S12. CD spectrum of compound **1**........ 17

Figure S13. ^{1}H NMR spectrum of compound **2** ($CDCl_3$)........ 18

Figure S14. ^{13}C NMR spectrum of compound **2** ($CDCl_3$)........ 19

Figure S15. DEPT 90 ^{13}C NMR spectrum of compound **2** ($CDCl_3$)........ 20

Figure S16. DEPT 135 ^{13}C NMR spectrum of compound **2** ($CDCl_3$)........ 21

Figure S17. HSQC NMR spectrum of compound **2** ($CDCl_3$)........ 22

Figure S18. ^{1}H-^{1}H COSY NMR spectrum of compound **2** ($CDCl_3$)........ 23

Figure S19. HMBC NMR spectrum of compound **2** ($CDCl_3$)........ 24

Figure S20. NOESY NMR spectrum of compound **2** ($CDCl_3$)........ 25

Figure S21. HRESI MS spectrum of compound **2**........ 26

Figure S22. UV spectrum of compound **2** 27

Figure S23. IR spectrum of compound **2** 28

Figure S24. CD spectrum of compound **2** 29

Figure S25. ^{1}H NMR spectrum of compound **3** ($CDCl_3$) 30

Figure S26. ^{13}C NMR spectrum of compound **3** ($CDCl_3$) 31

Figure S27. DEPT 90 ^{13}C NMR spectrum of compound **3** ($CDCl_3$) 32

Figure S28. DEPT 135 ^{13}C NMR spectrum of compound **3** (CDCl3) 33

Figure S29. HSQC NMR spectrum of compound **3** ($CDCl_3$) 34

Figure S30. ^{1}H-^{1}H COSY NMR spectrum of compound **3** ($CDCl_3$) 35

Figure S31. HMBC NMR spectrum of compound **3** ($CDCl_3$) 36

Figure S32. NOESY NMR spectrum of compound **3** ($CDCl_3$) 37

Figure S33. HRESIMS spectrum of compound **3** 38

Figure S34. UV spectrum of compound **3** 39

Figure S35. IR spectrum of compound **3** 40

Figure S36. CD spectrum of compound **3** 41

Figure S37. ^{1}H NMR spectrum of compound **4** (Pyridine-d_5) 42

Figure S38. ^{13}C NMR spectrum of compound **4** (Pyridine-d_5) 43

Figure S39. ^{1}H NMR spectrum of compound **5** ($CDCL_3$) 44

Figure S40. ^{13}C NMR spectrum of compound **5** ($CDCl_3$) 45

Figure S41. ^{1}H NMR spectrum of compound **6** ($CDCL_3$) 46

Figure S42. ^{13}C NMR spectrum of compound **6** ($CDCl_3$) 47

Figure S43. ^{1}H NMR spectrum of compound **7** ($CDCL_3$) 48

Figure S44. ^{13}C NMR spectrum of compound **7** ($CDCl_3$) 49

Figure S45. ^{1}H NMR spectrum of compound **8** ($CDCL_3$) 50

Figure S46. ^{13}C NMR spectrum of compound **8** ($CDCl_3$) 51

Table S1 ^{1}H and ^{13}C NMR spectroscopic data of compound (**4-7**)

(δ in ppm, J in Hz) recorded at 600 MHz in $CDCl_3$.

Num.	**4**		**5**		**6**		**7**	
	δ_H	δ_C	δ_H	δ_C	δ_H	δ_C	δ_H	δ_C
1α	2.19 (dt, 15, 5)	49.0		72.6	1.38 (m),	41.0	3.33 (dd, 11.7, 4.0)	79.4
1β					1.06 (m)			
2α	2.47 (dd, 15, 5)	35.2	1.50 (m)	21.9	1.54 (m)	20.1	1.62 (overlap)	26.7
2β	2.36 (dd, 15, 15)		1.57 (m)		1.54 (m)		1.88 (ddd, 15.7, 12.7, 4.0)	
3α		199.9	5.35 (m)	122.0	1.78 (dd, 12.5, 3.2, 1.6)	43.4	1.72 (m)	39.7
3β					1.35 (m)		1.72 (m)	
4		132.3		133.7		72.3		71.6
5α	6.64 s	154.6	2.00 (m)	26.6	1.19 (m)	54.8	1.47 (d, 3.3)	44.6
5β			1.92 (m)					
6α		71.9	1.58 (m)	48.0	1.59 (m)	22.5	1.45 (dd, 5.6, 3.1)	29.0
6β					1.28 (m)		1.45 (dd, 5.6, 3.1)	
7α		47.3		49.2	1.34 (m)	49.9		73.8
7β	2.53 dd (15 5)		1.06 (m)					
8α	1.97 (m)	35.3	0.97 (m)	24.0	1.91 (ddd, 12.6, 5.3, 2.4)	21.5	1.56 (overlap)	29.3
8β	1.64 (m)		1.44 (m)		1.02 (m)		1.56 (overlap)	
9α		70.5	1.51 (overlap)	31.1	1.44 (dt, 12.5, 3.2)	44.6	1.68 (dd, 4.0, 2.5)	34.6
9β	3.46 (dt, 15, 5)		0.97 (m)		1.15 (m)		1.39 (m)	
10	2.12 (m)	35.9	1.51 (overlap)	41.8		34.5		38.8
11		145.4	1.85 (m)	26.8	1.19 (s)	27.1	0.99 (s)	11.6
12a	4.74(s)	21.4	0.78 (d 7.0)	21.5		72.9	1.61 (overlap)	39.2
12b	4.86 (s)							
13	1.83 (s)	113.0	0.71 (d 6.9)	15.1	1.20 (s)	27.29	0.96 (d, 2.4)	16.8
14	1.00 (d, 10)	13.5	0.87 (d 6.6)	15.1	1.11 (s)	22.61	0.95 (d, 2.4)	17.0
15	1.71 (s)	14.0	1.61 (s)	23.4	0.86 (s)	18.65	1.14 (s)	29.8

Table S2. ^{1}H and ^{13}C NMR spectroscopic data of cytochalasin H (**8**)

(δ in ppm, J in Hz) recorded at 600 MHz in $CDCl_3$.

Num.	δ_H	δ_C	Num.	δ_H	δ_C
1		174.2	16	1.79 (m)	28.5
3	3.26 (m)	53.8	17*α*	1.89 (dd 14.3 2.8)	53.7
4	2.13 (m)	50.5	17*β*	1.57 (m)	
5	2.78 (m) CH	32.9	18		74.4
6		147.9	19	5.54 (dd 16.2 2.4)	138.1
7	3.83 (d 10.6)	69.7	20	5.87 (dd 16.5 2.5)	126.0
8	2.94 (t 10.3)	47.2	21	5.56 (m)	77.5
9		51.8	22	1.05 (d 6.4)	26.5
10*α*	2.87 (dd 13.5 4.4)	45.7	23	1.35 (s)	31.2
10*β*	2.65 (dd 13.4 9.8)		24		170.2
11	1.01 (d 6.7)	14.1	25	2.25 (s)	20.9
12a	5.12 (s)	114.2	26		137.4
12b	5.36 (s)		27	7.15 (d 7.4)	129.0
13	5.74 (dd 15.5 9.7)	127.1	28	7.32 (m)	129.0
14	5.41 (ddd 15.3 10.2 4.8)	138.8	29	7.25 (m)	127.1
15*α*	2.04 (m)	42.8	30	7.32 (m)	129.0
15*β*	1.81 (m)		31	7.15 (d 7.4)	129.0

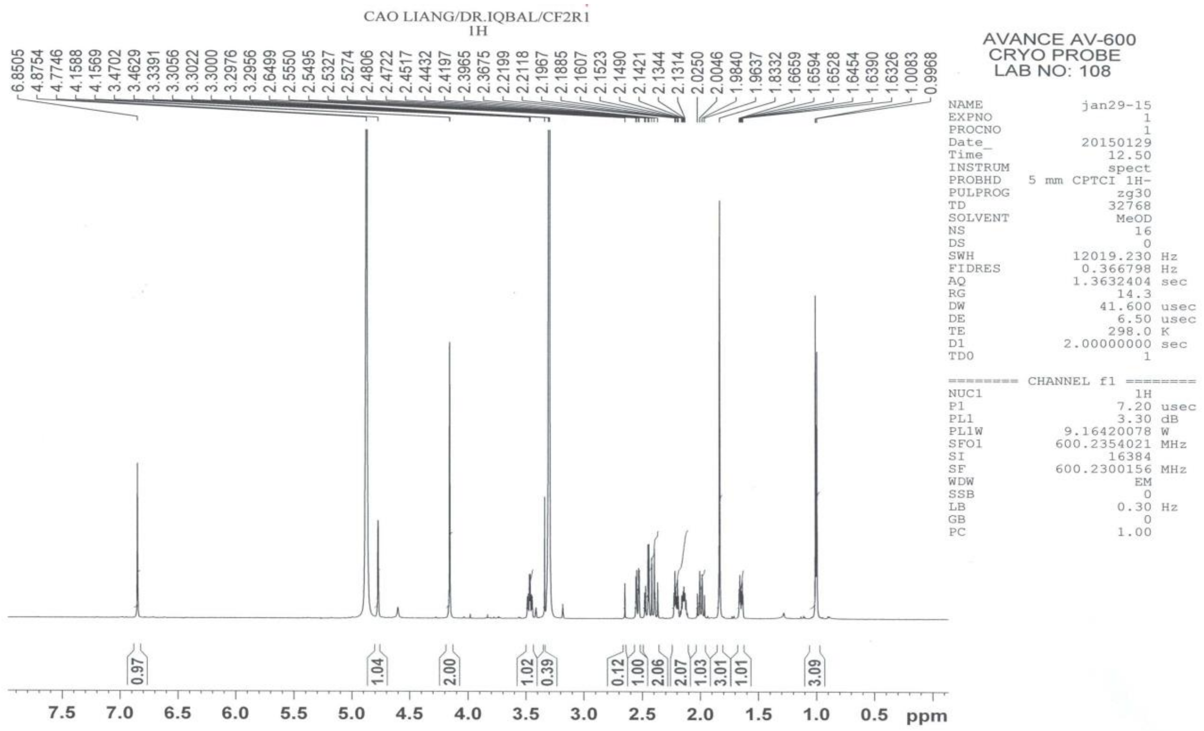

Figure S1. ^{1}H NMR spectrum of compound **1** (CDCl3)

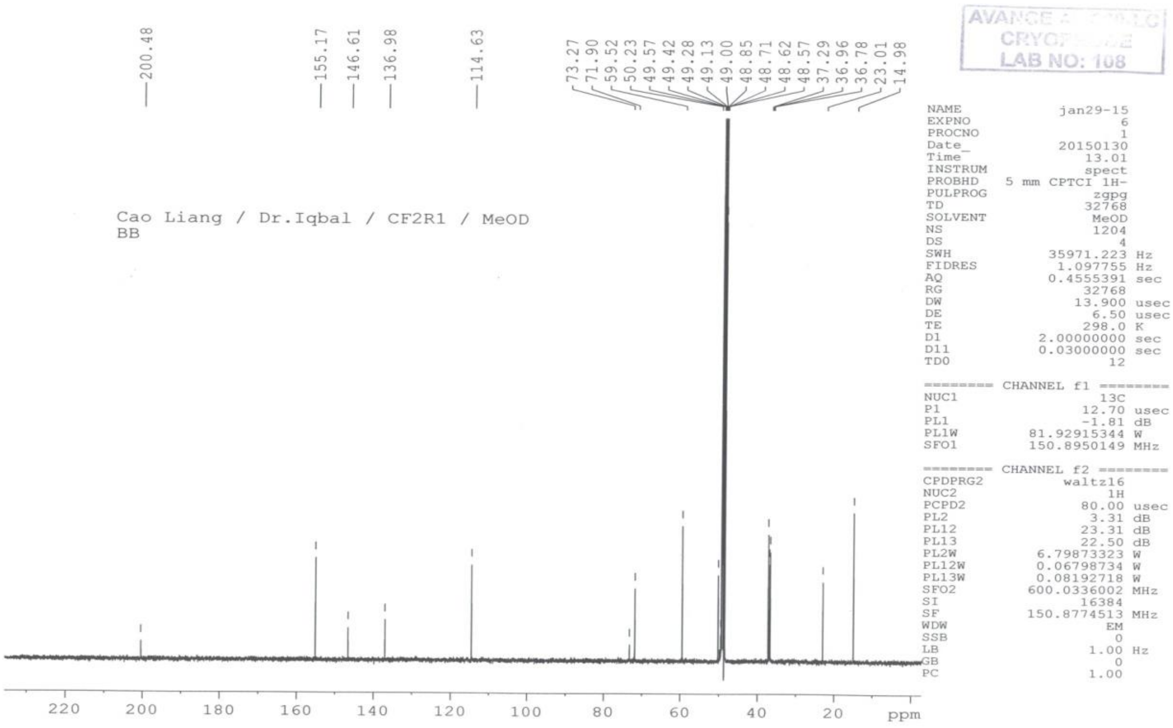

Figure S2. ^{13}C NMR spectrum of compound **1** ($CDCl_3$)

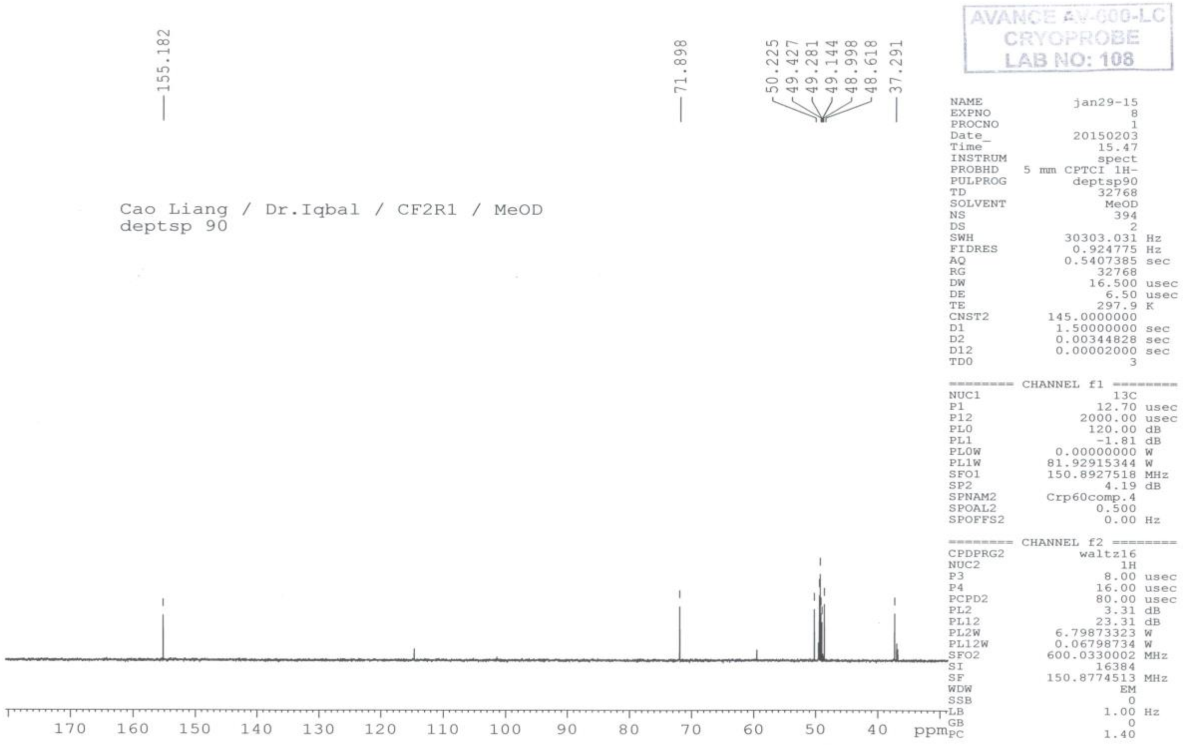

Figure S3. DEPT 90 ^{13}C NMR spectrum of compound **1** ($CDCl_3$)

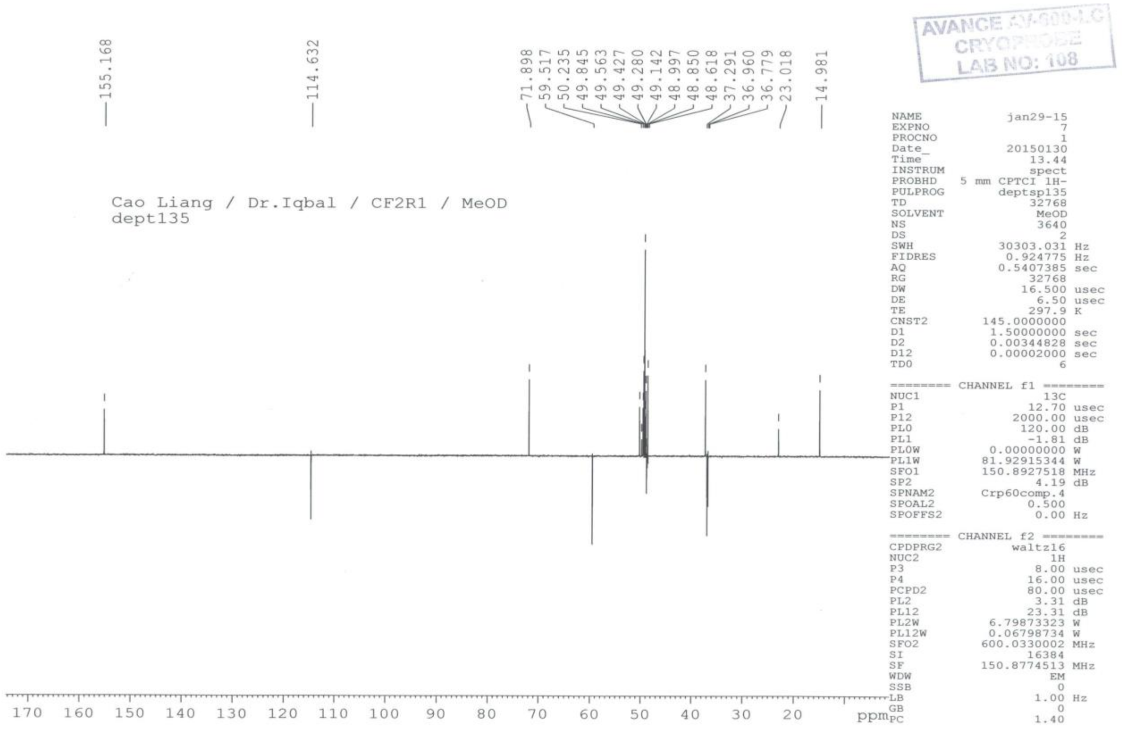

Figure S4. DEPT 135 ^{13}C NMR spectrum of compound **1** ($CDCl_3$)

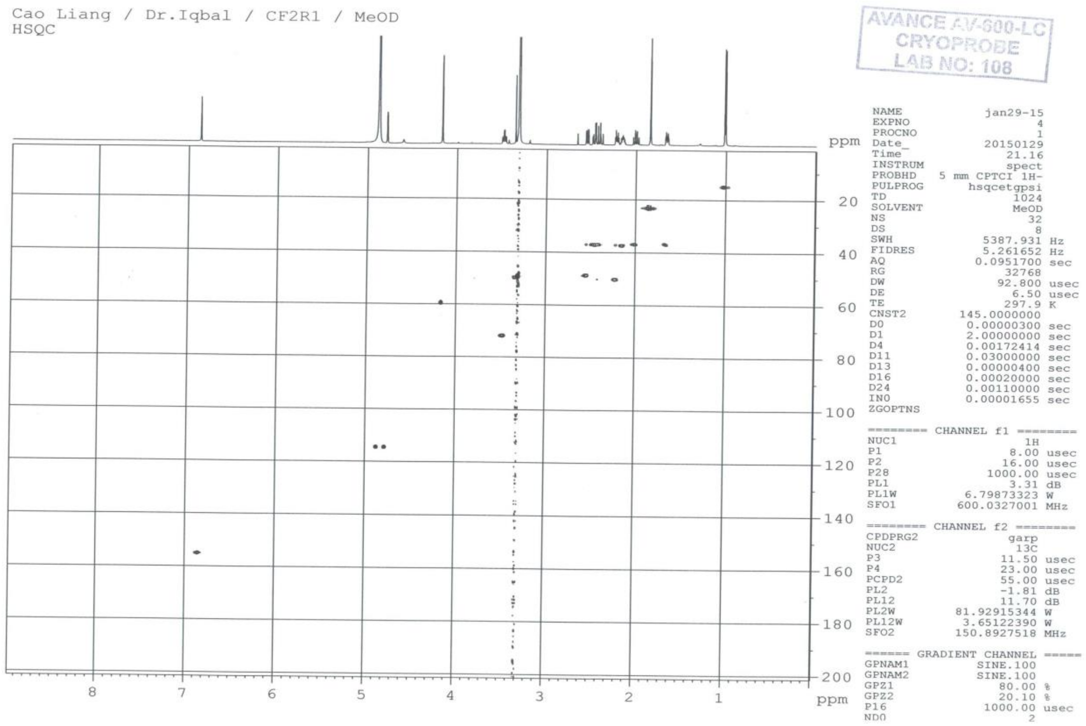

Figure S5. HSQC NMR spectrum of compound **1** ($CDCl_3$)

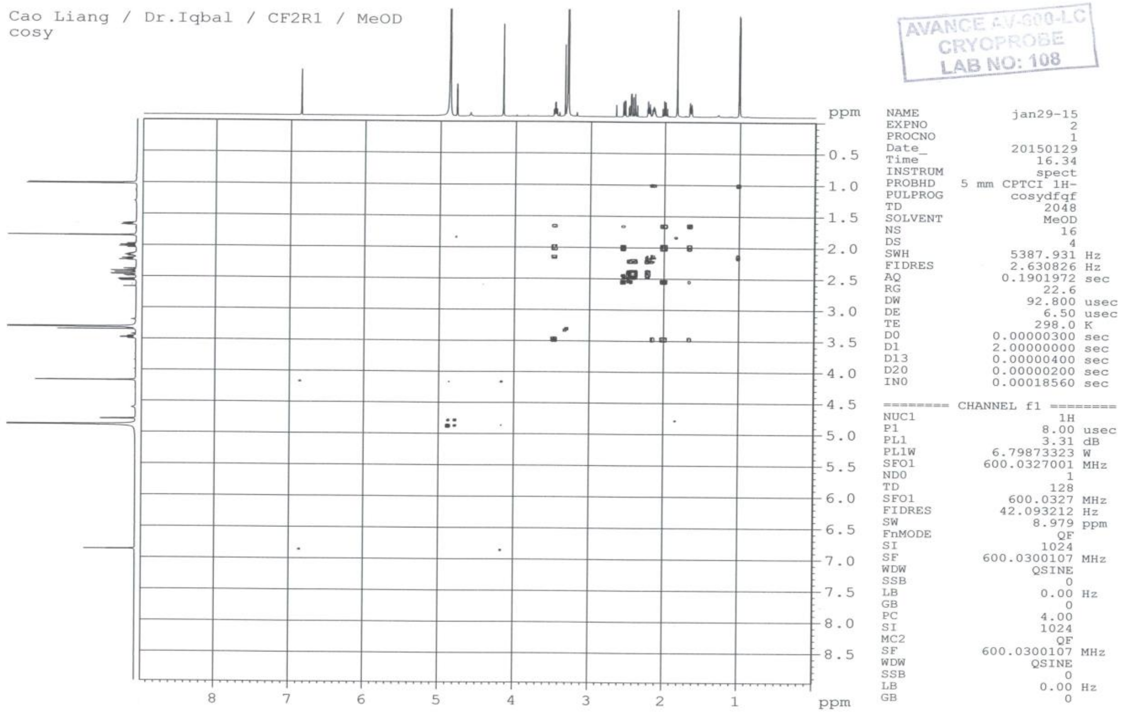

Figure S6. ^{1}H-^{1}H COSY NMR spectrum of compound **1** ($CDCl_3$)

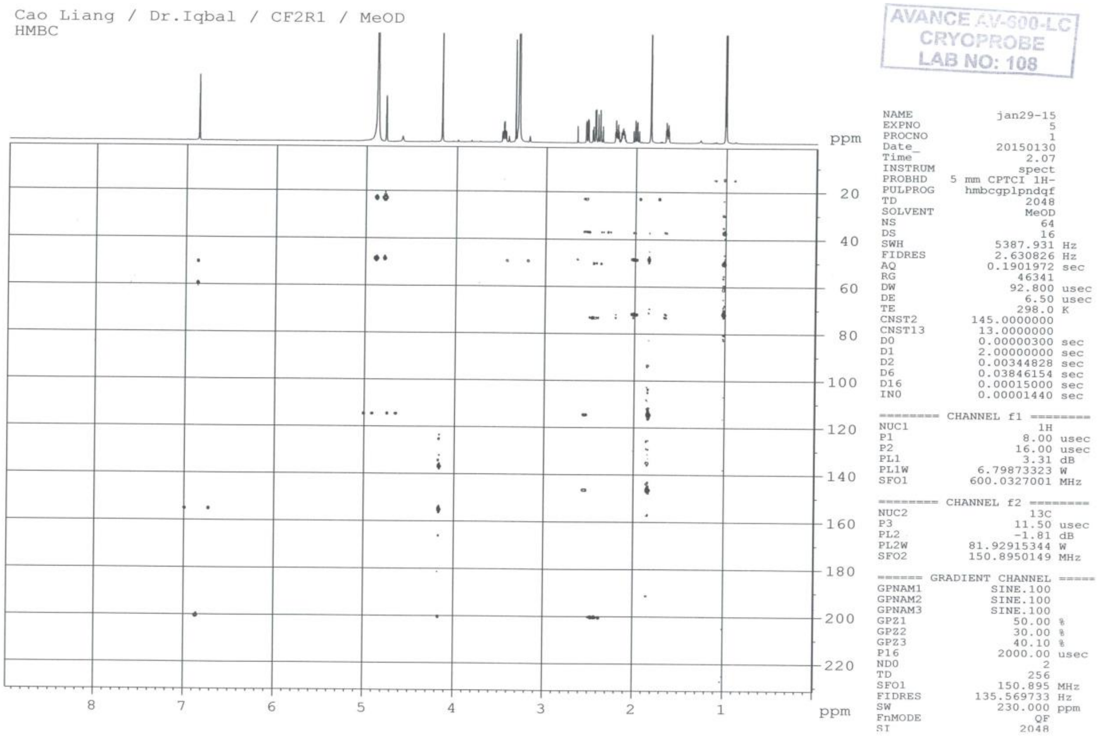

Figure S7. HMBC NMR spectrum of compound **1** ($CDCl_3$)

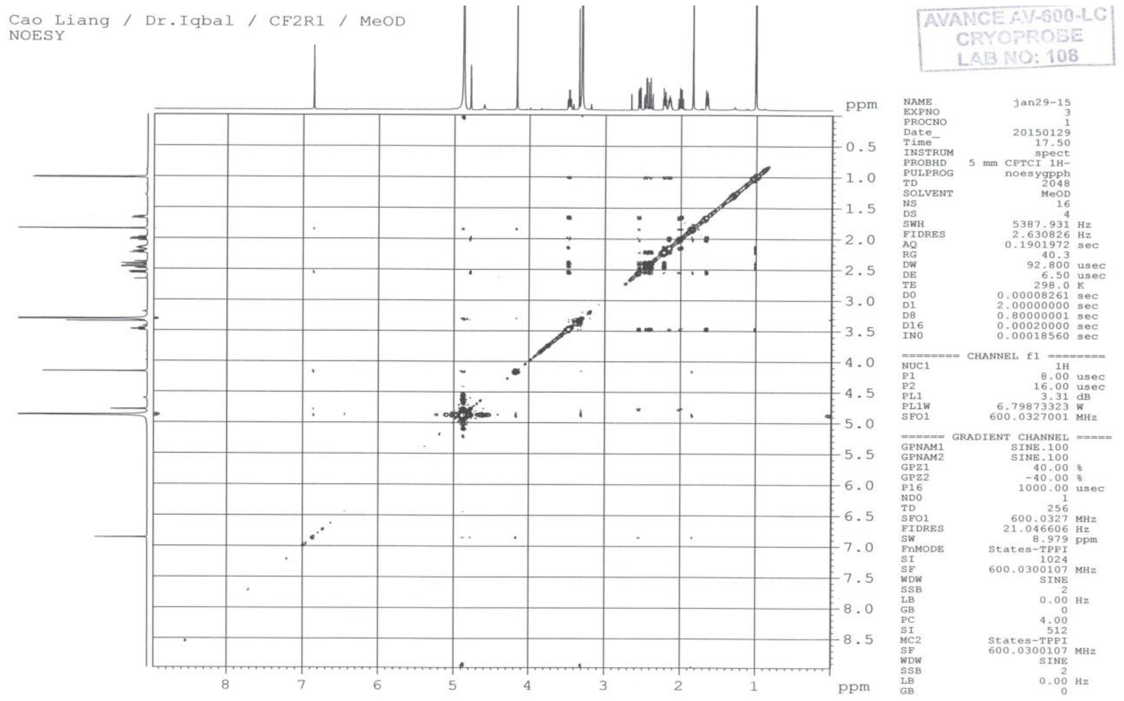

Figure S8. NOESY NMR spectrum of compound **1** ($CDCl_3$)

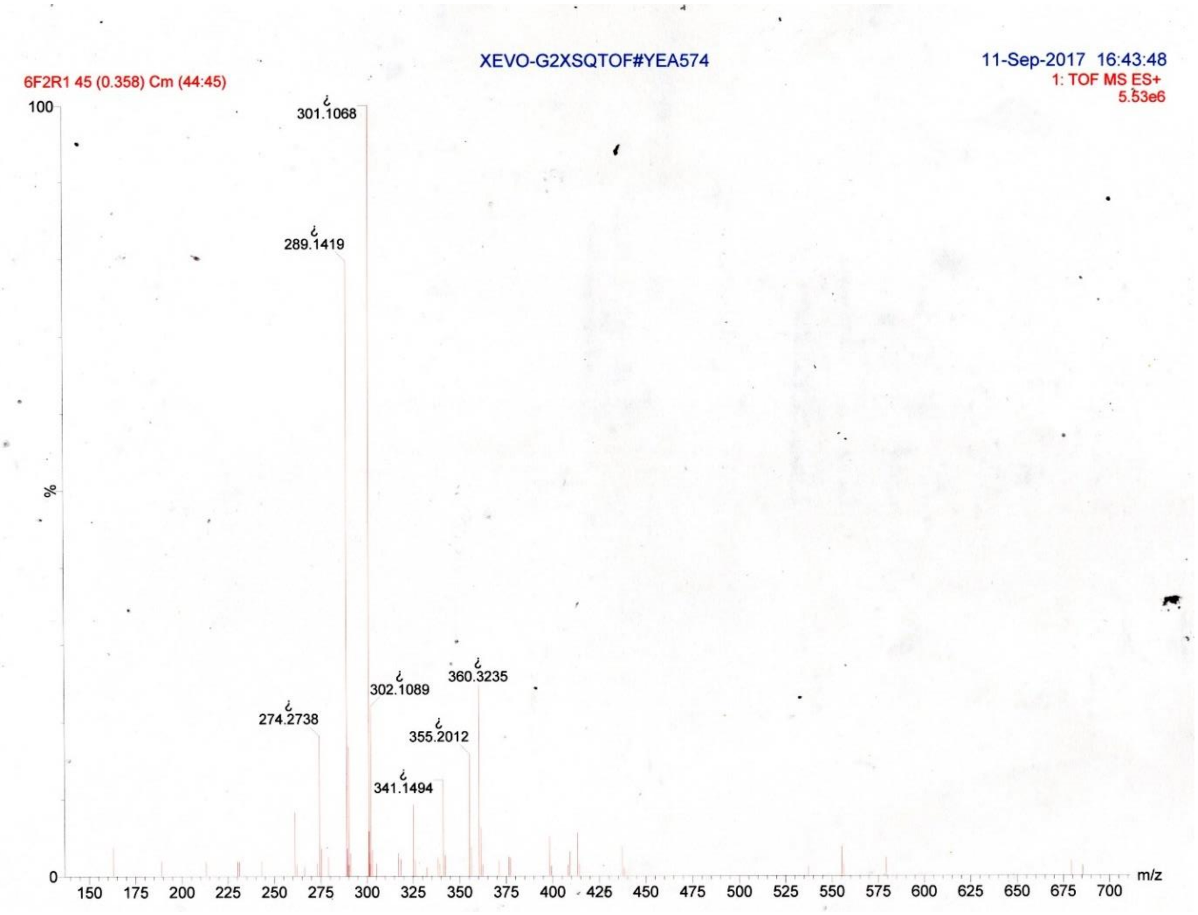

Figure S9. HRESIMS spectrum of compound **1**

THERMO ELECTRON ~ VISIONpro SOFTWARE V4.10

Operator Name	ARSHAD ALAM	Date of Report	4/28/2015
Department	Analytical laboratory # 004 TWC	Time of Report	3:48:56PM
Organization	ICCBS.Karachi University.		
Information	CaoLiang./ Porf. Dr. M.Iqbal.		

Scan Graph

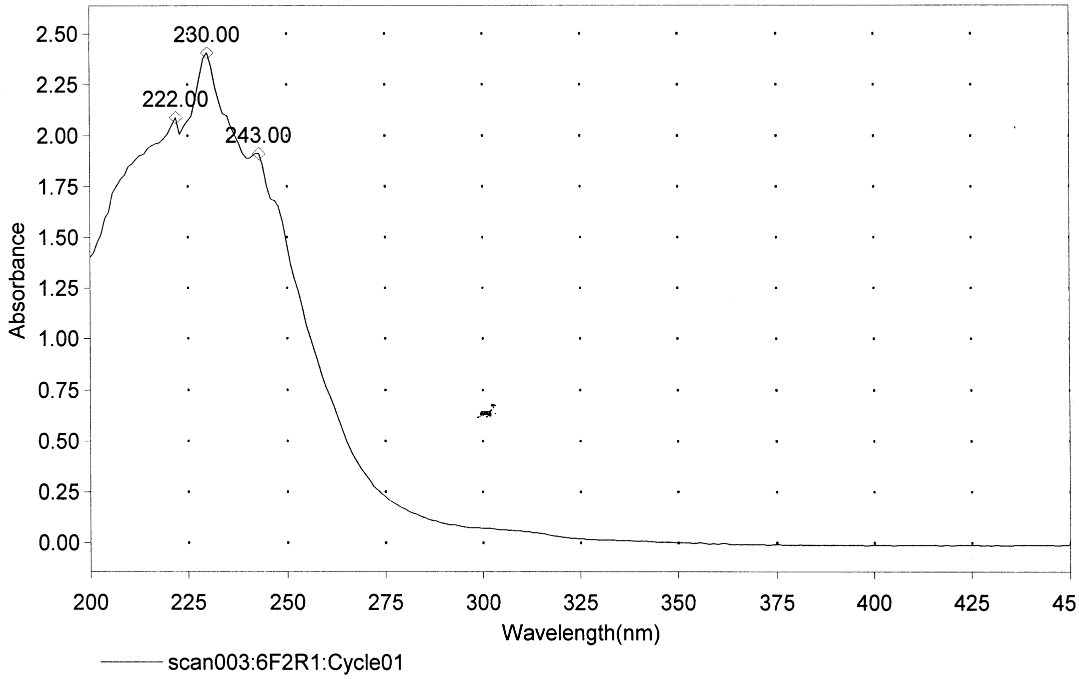

Results Table - 6- F2R1.sre,6F2R1,Cycle01

nm	A	**Peak Pick Method**
222.00	2.089	Find 8 Peaks Above -3.0000 A
230.00	2.404	Start Wavelength 200.00 nm
243.00	1.912	Stop Wavelength 250.00 nm
		Sort By Wavelength

Sensitivity	Manual
Rising Points	1
Falling Points	1
Min. Change	0.0000

Figure S10. UV spectrum of compound **1**

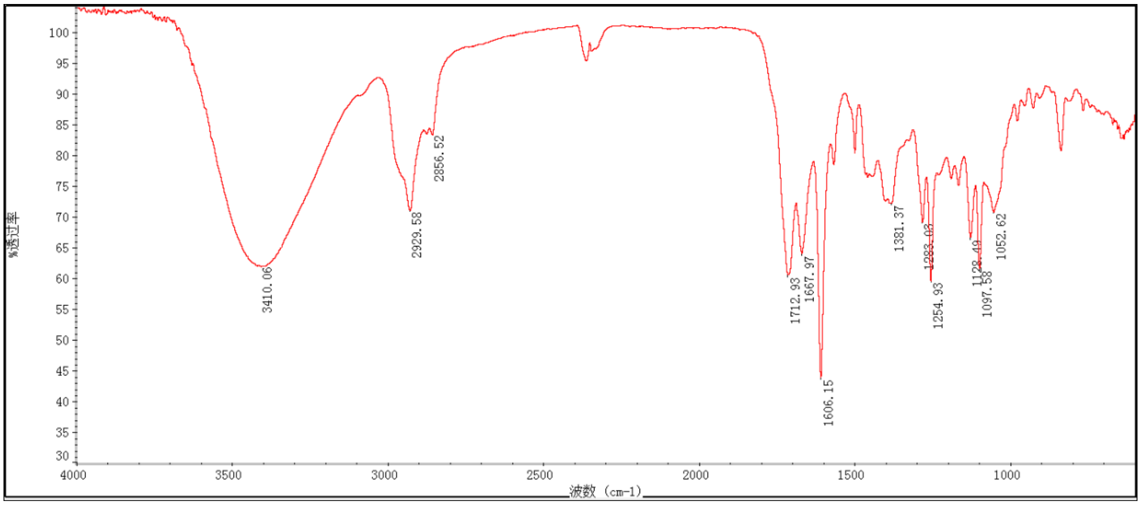

Figure S11. IR spectrum of compound **1**

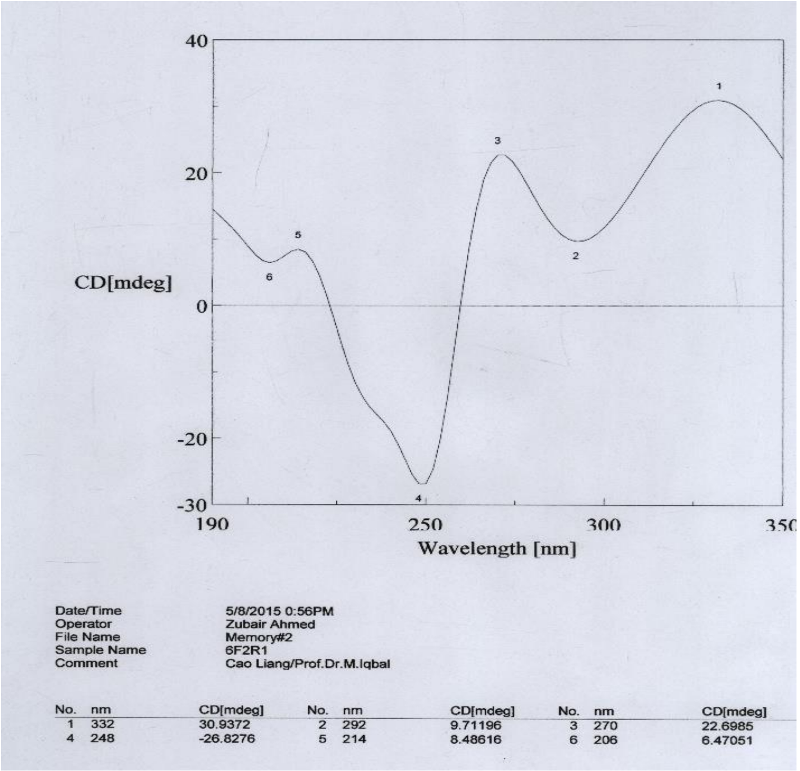

Figure S12. CD spectrum of compound **1**

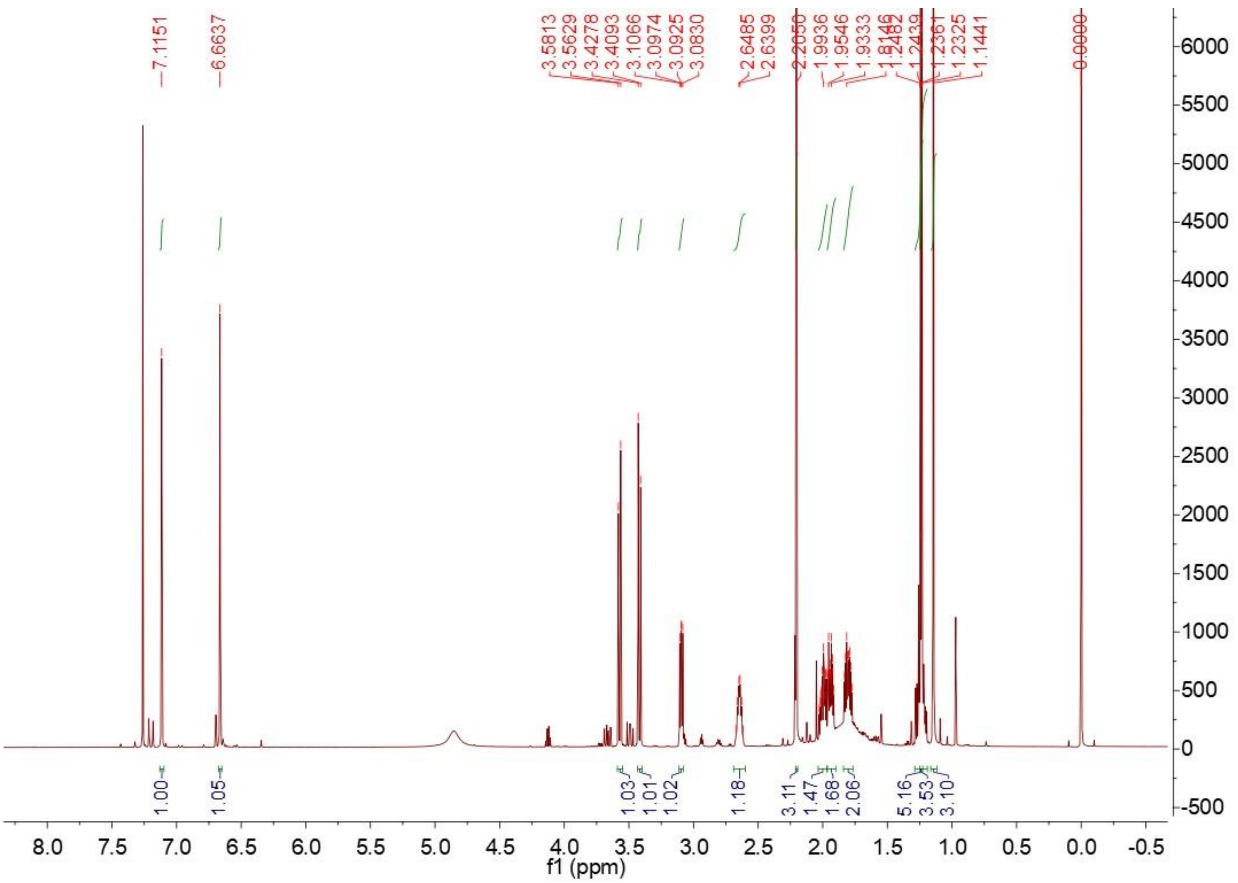

Figure S13. ^{1}H NMR spectrum of compound **2** ($CDCl_3$)

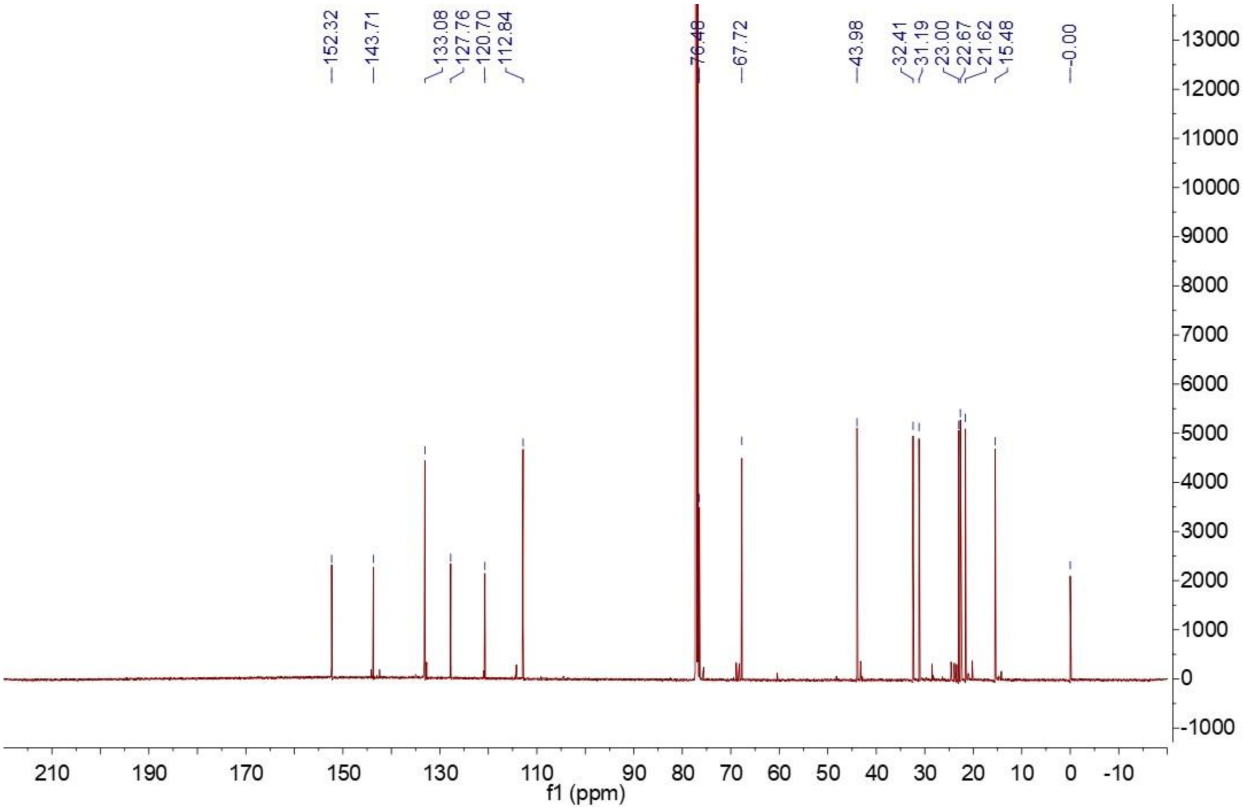

Figure S14. ^{13}C NMR spectrum of compound **2** ($CDCl_3$)

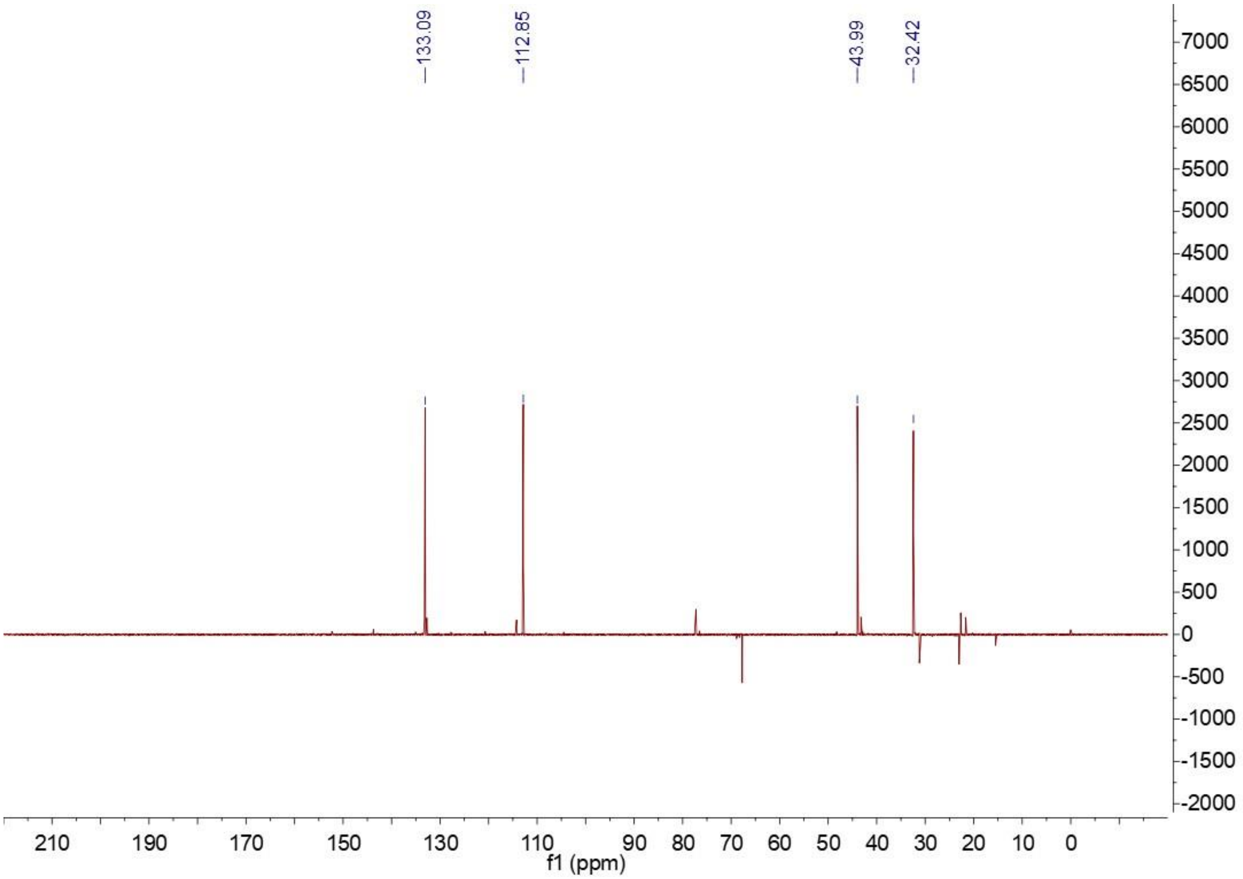

Figure S15. DEPT 90 ^{13}C NMR spectrum of compound **2** ($CDCl_3$)

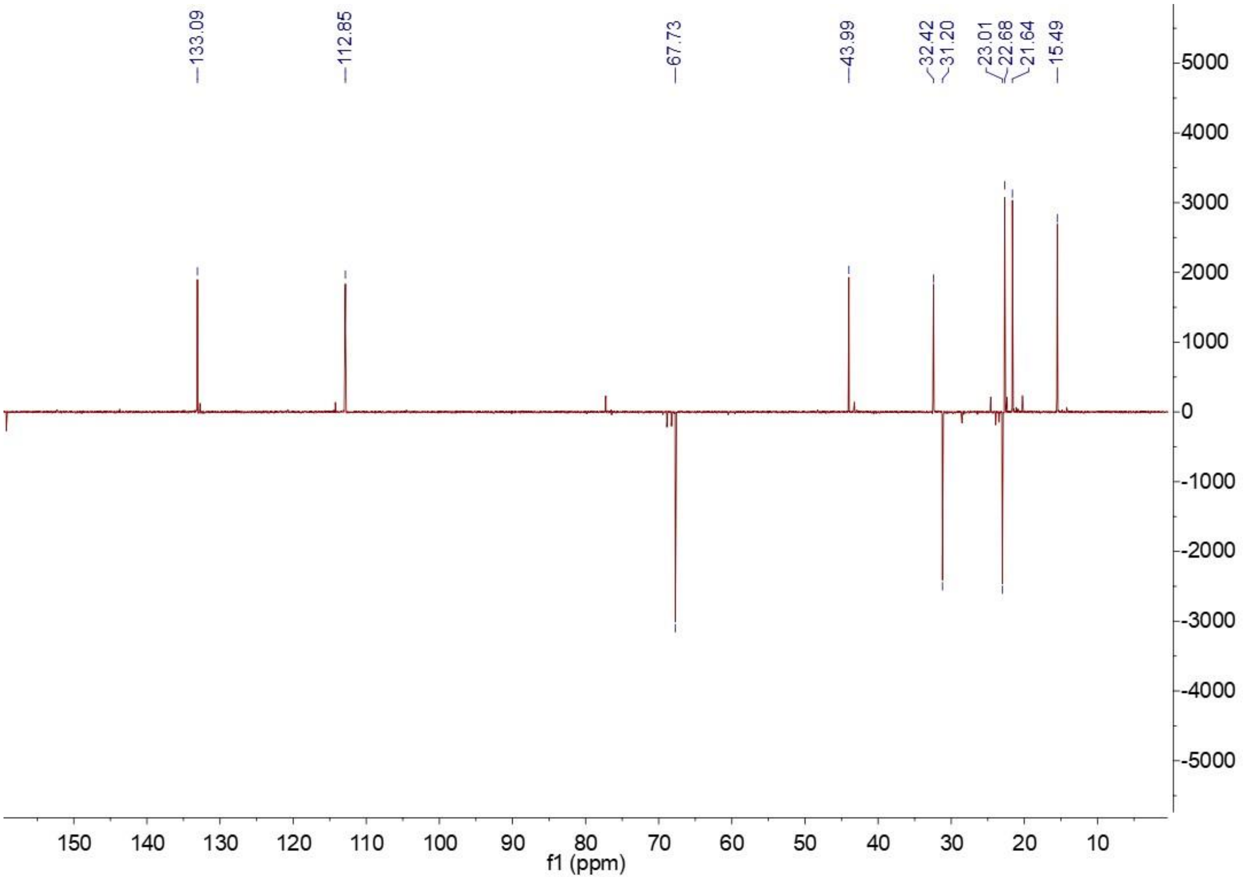

Figure S16. DEPT 135 ^{13}C NMR spectrum of compound **2** ($CDCl_3$)

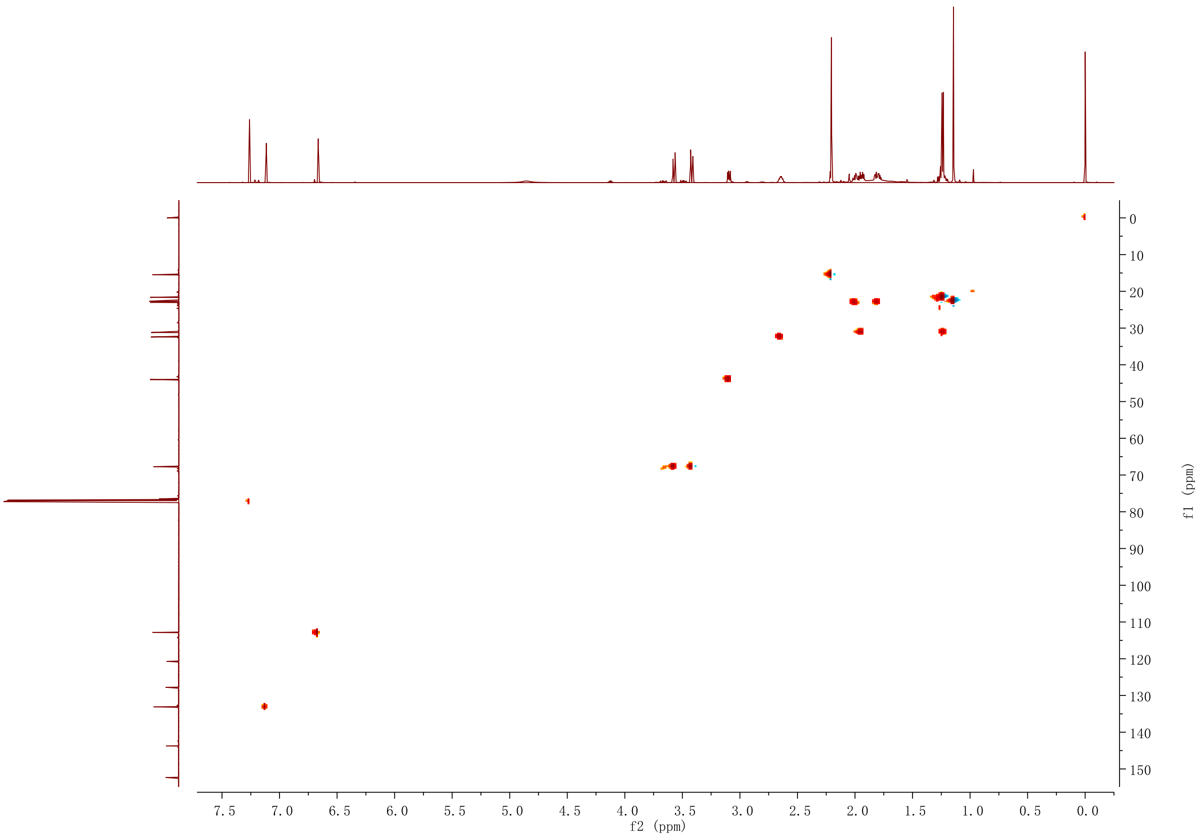

Figure S17. HSQC NMR spectrum of compound **2** ($CDCl_3$)

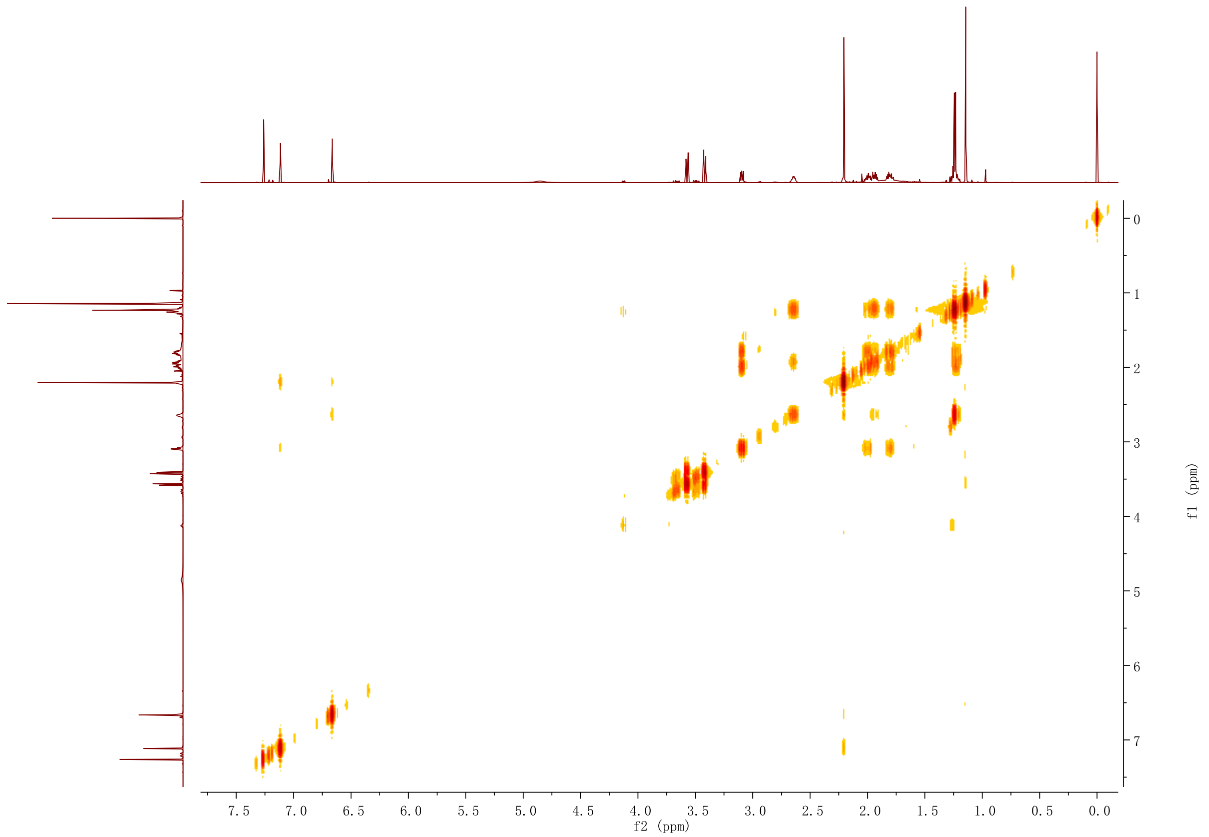

Figure S18. ^{1}H-^{1}H COSY NMR spectrum of compound **2** ($CDCl_3$)

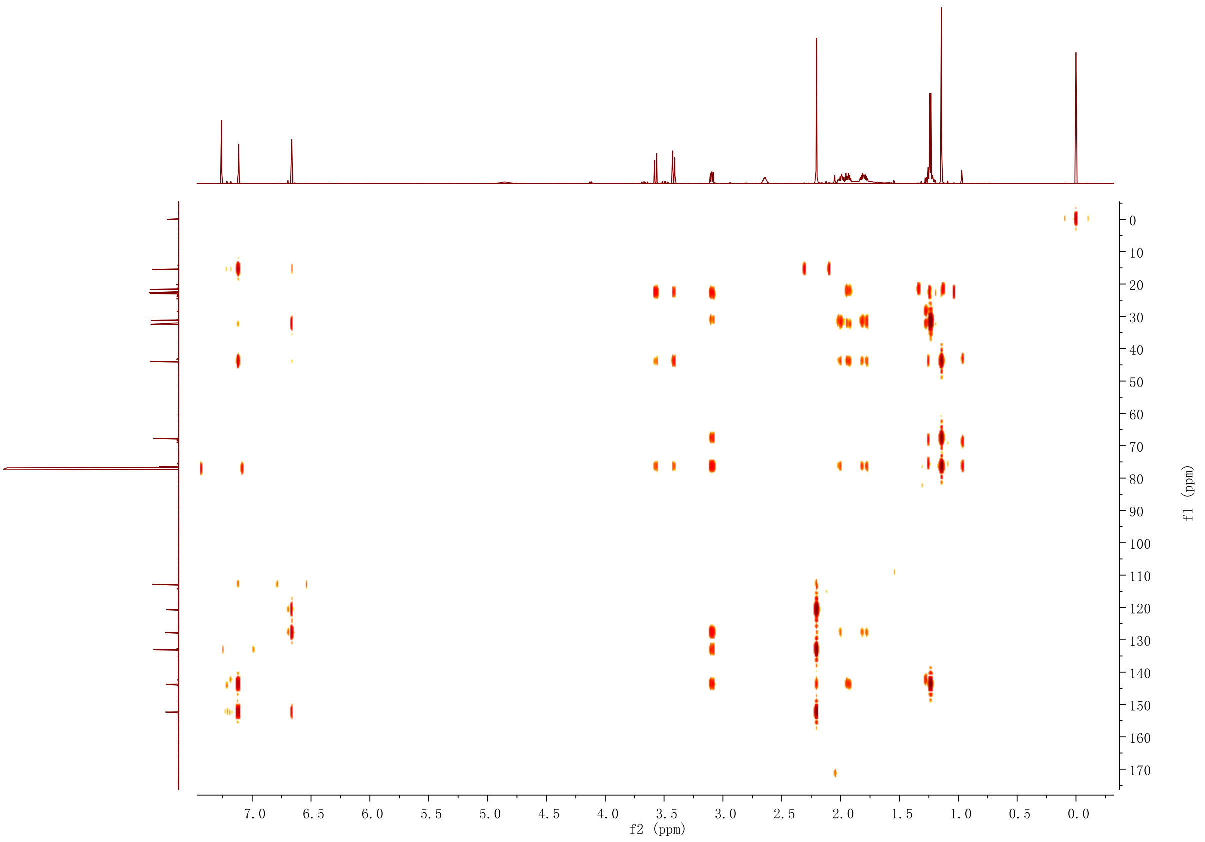

Figure S19. HMBC NMR spectrum of compound **2** ($CDCl_3$)

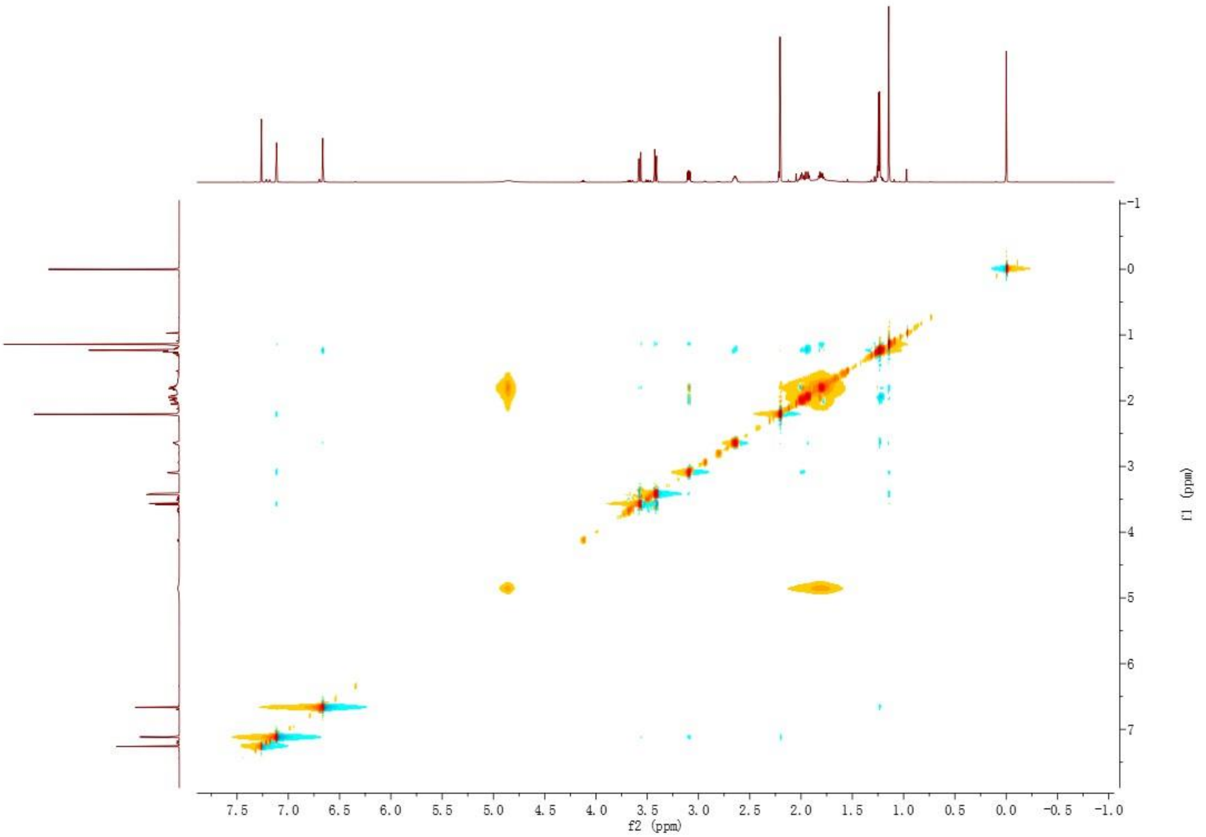

Figure S20. NOESY NMR spectrum of compound **2** ($CDCl_3$)

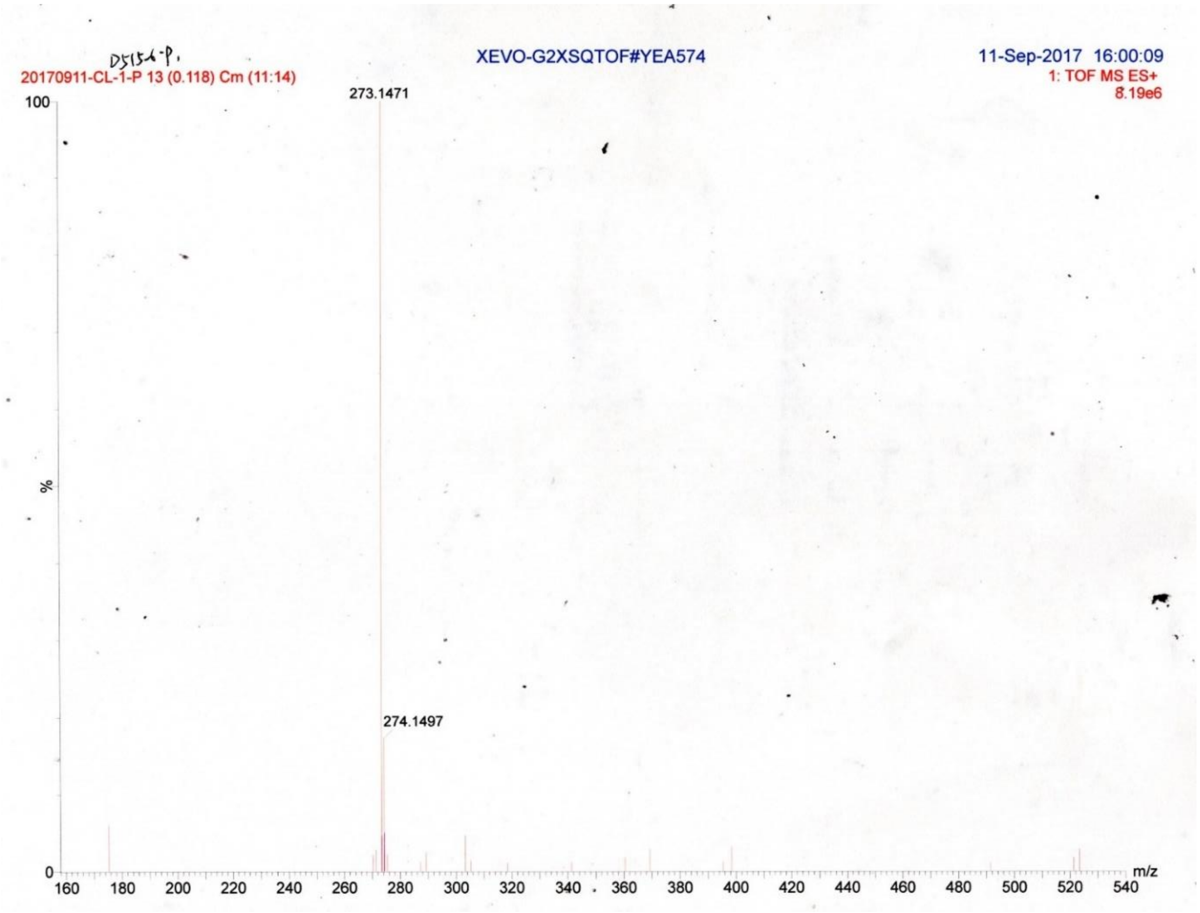

Figure S21. HRESI MS spectrum of compound **2**

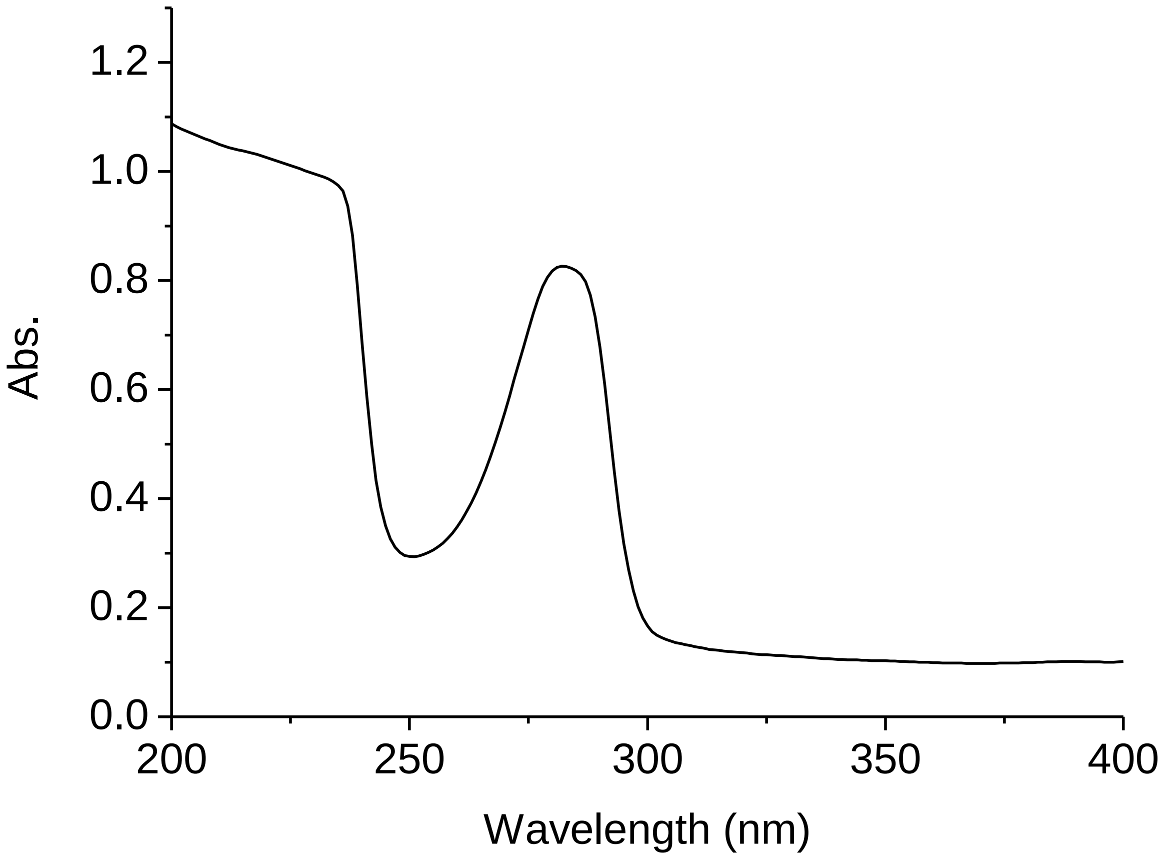

Figure S22. UV spectrum of compound **2**

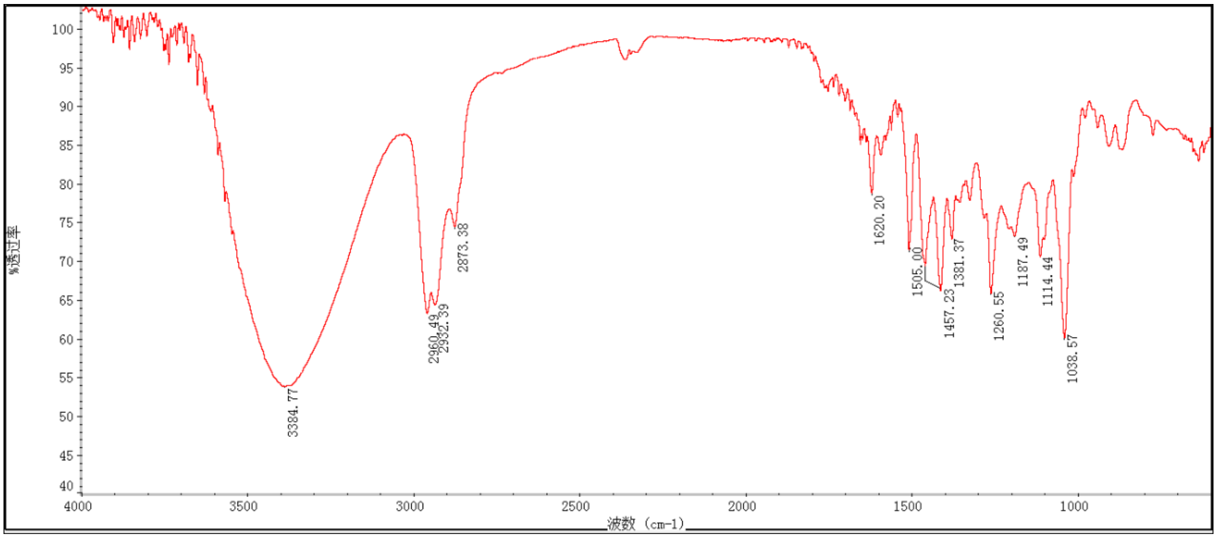

Figure S23. IR spectrum of compound **2**

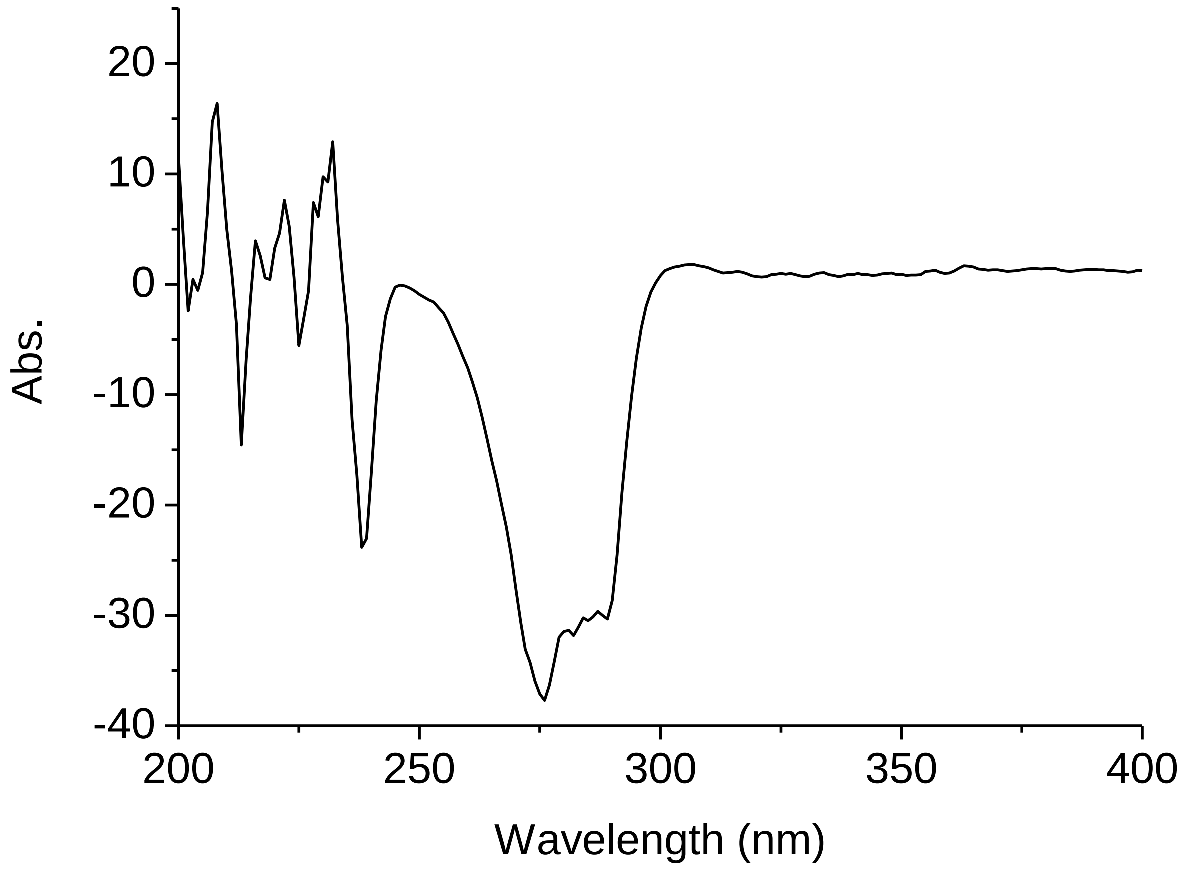

Figure S24. CD spectrum of compound **2**

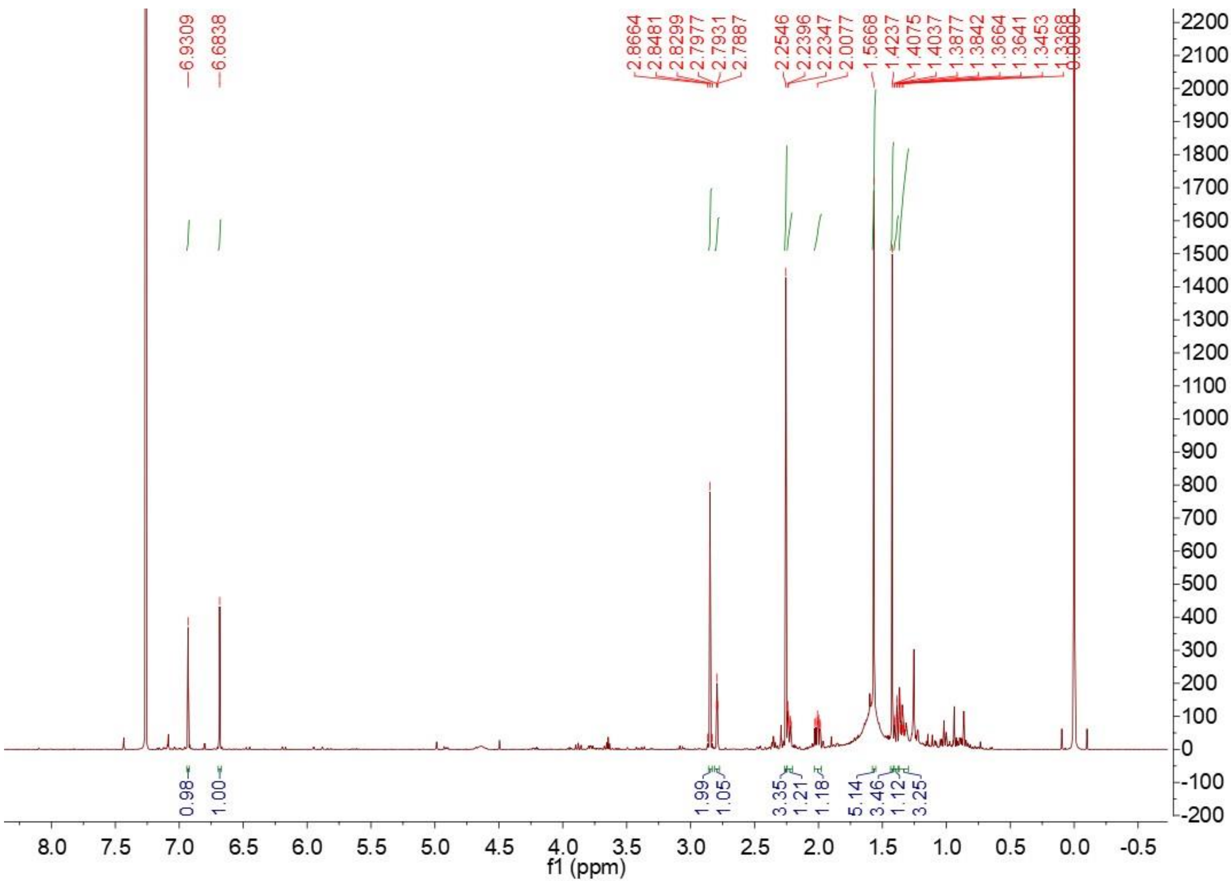

Figure S25. ^{1}H NMR spectrum of compound **3** ($CDCl_3$)

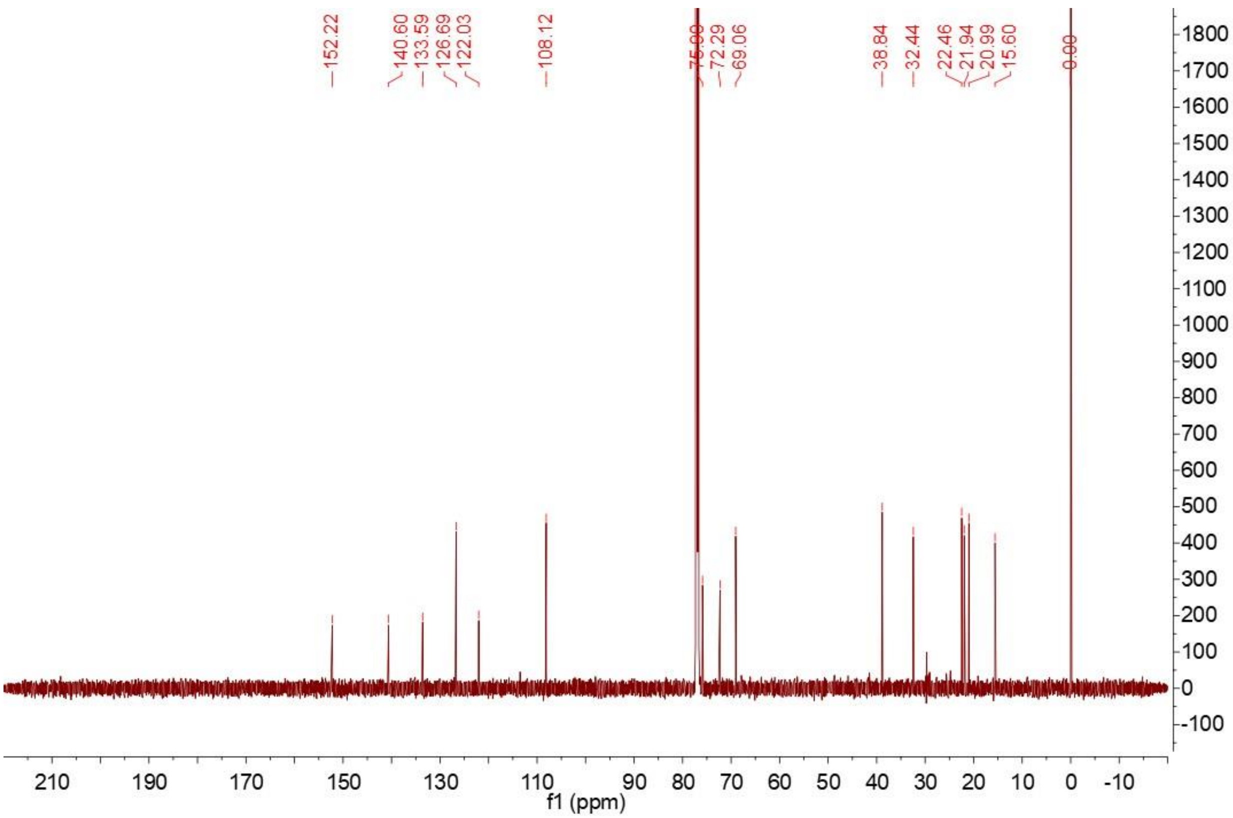

Figure S26. ^{13}C NMR spectrum of compound **3** ($CDCl_3$)

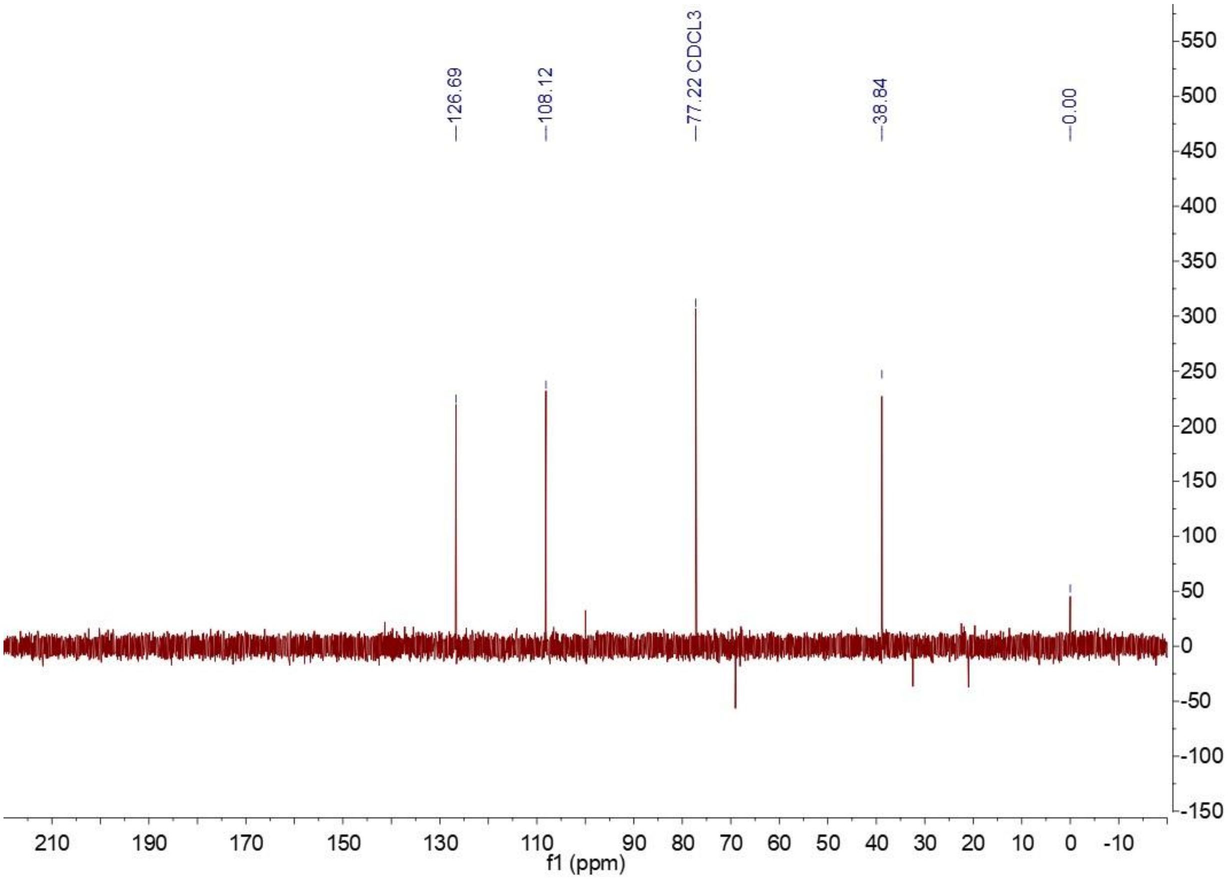

Figure S27. DEPT 90 ^{13}C NMR spectrum of compound **3** ($CDCl_3$)

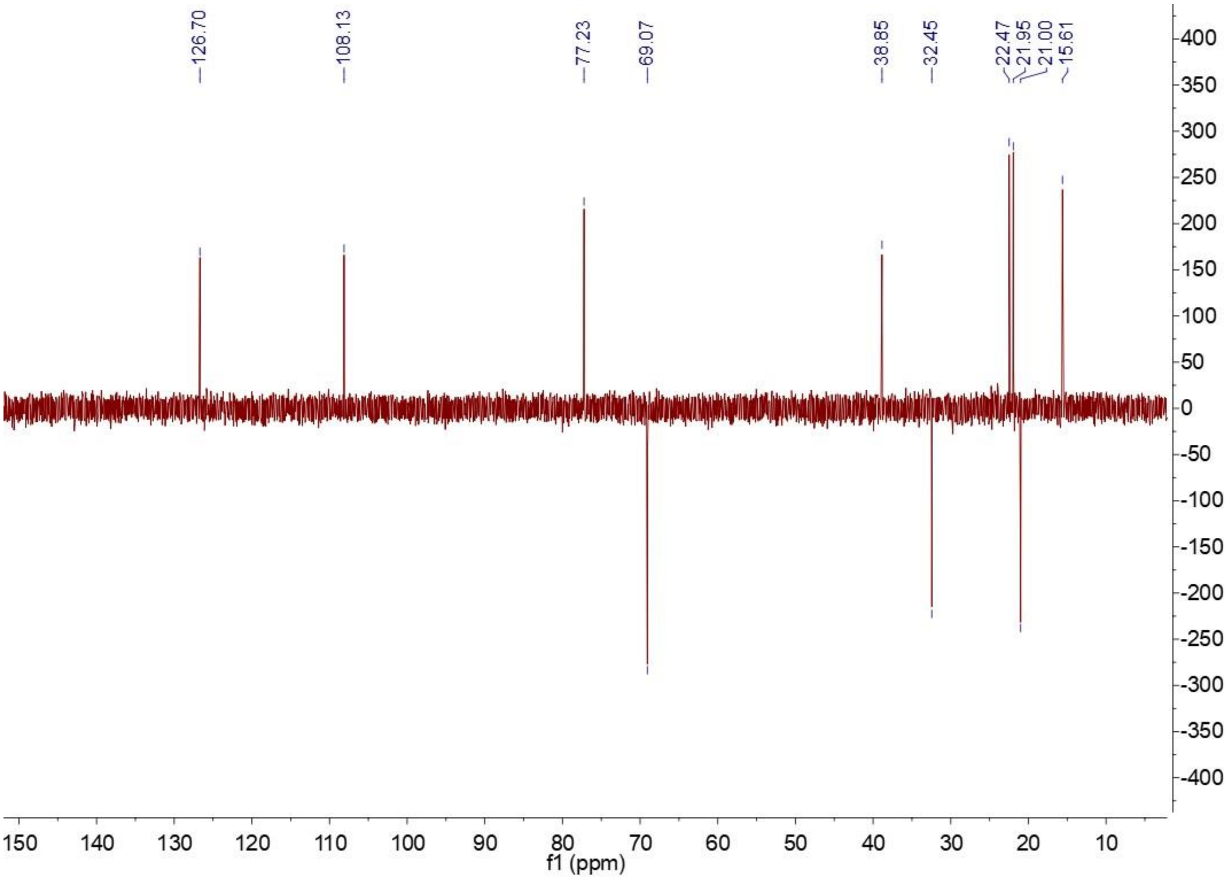

Figure S28. DEPT 135 ^{13}C NMR spectrum of compound **3** (CDCl3)

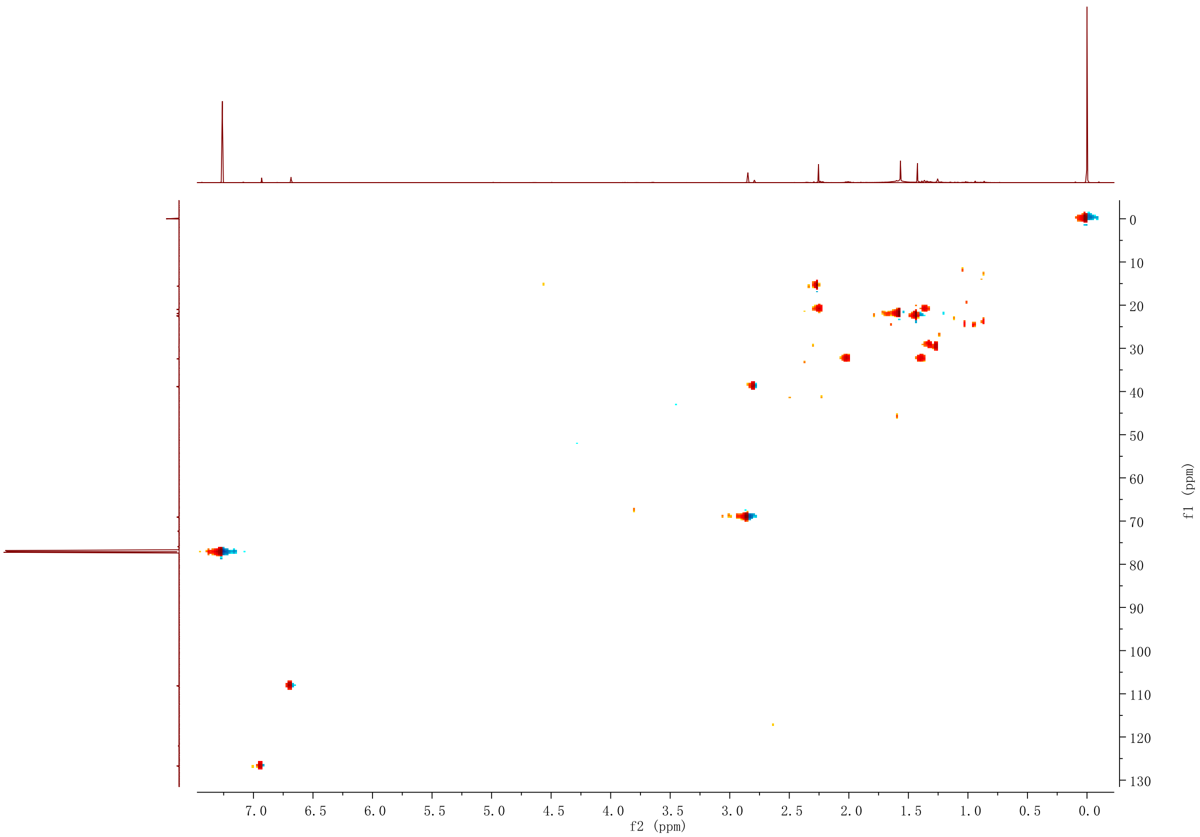

Figure S29. HSQC NMR spectrum of compound **3** ($CDCl_3$)

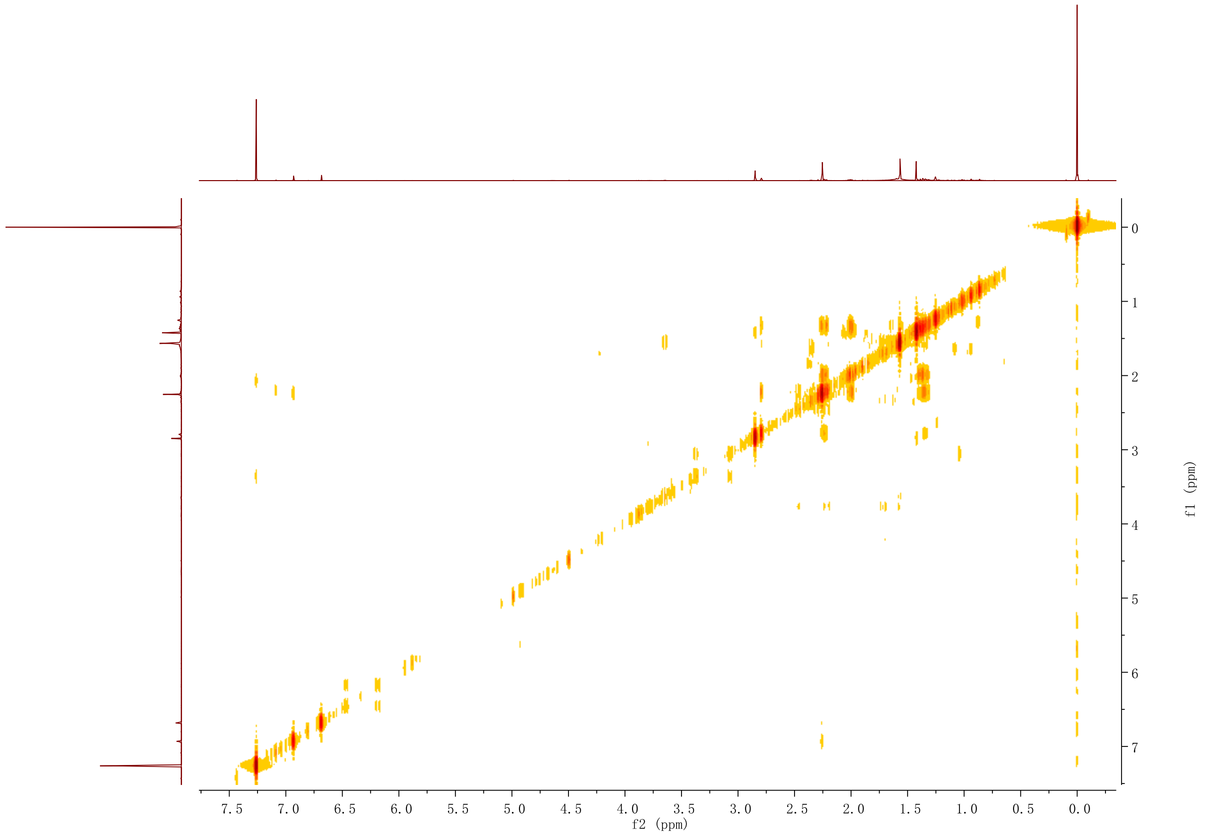

Figure S30. ^{1}H-^{1}H COSY NMR spectrum of compound **3** ($CDCl_3$)

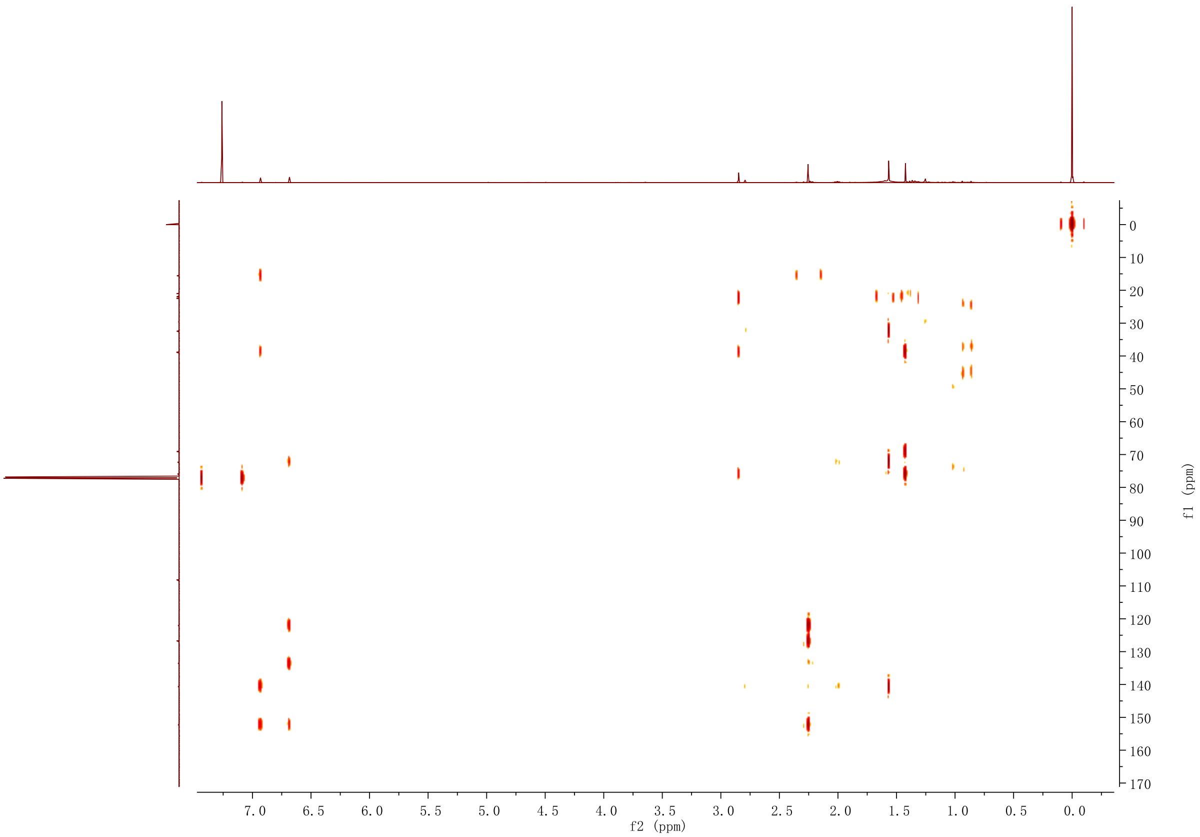

Figure S31. HMBC NMR spectrum of compound **3** ($CDCl_3$)

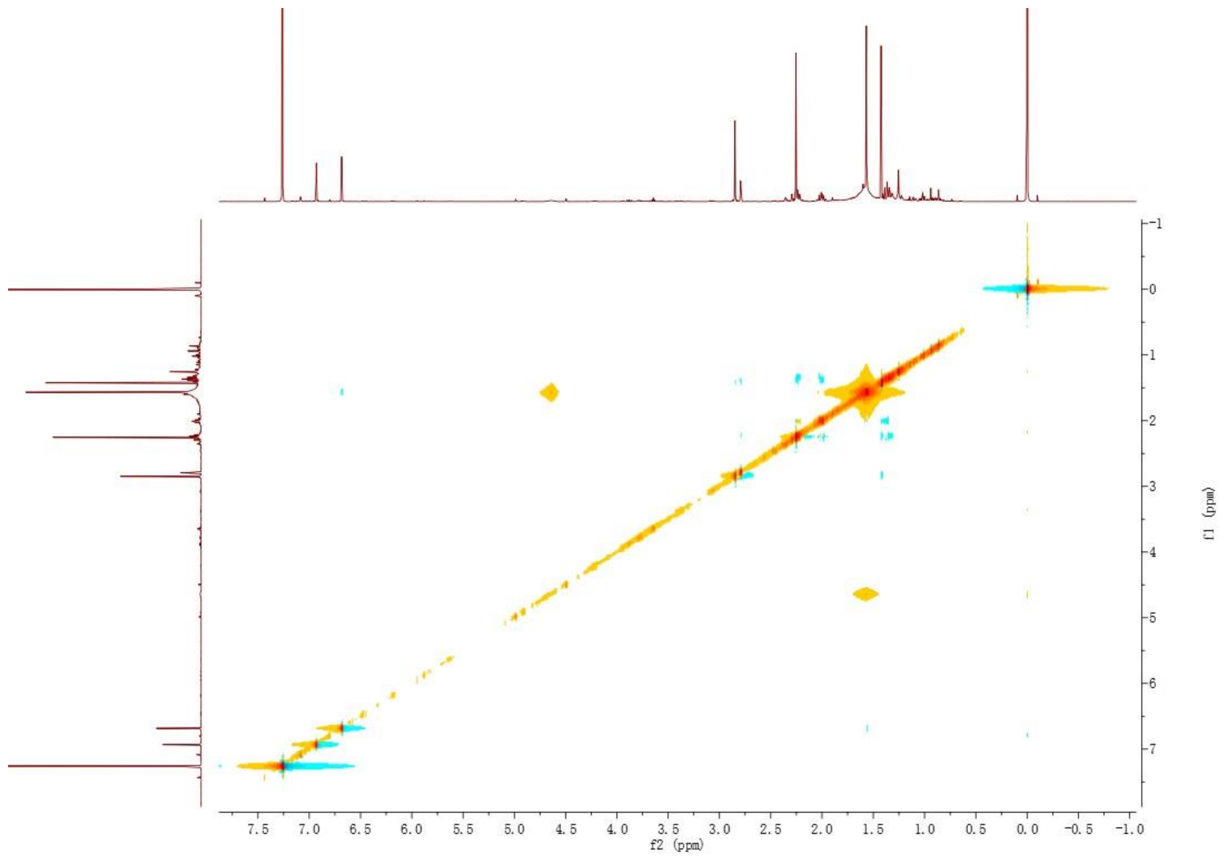

Figure S32. NOESY NMR spectrum of compound **3** ($CDCl_3$)

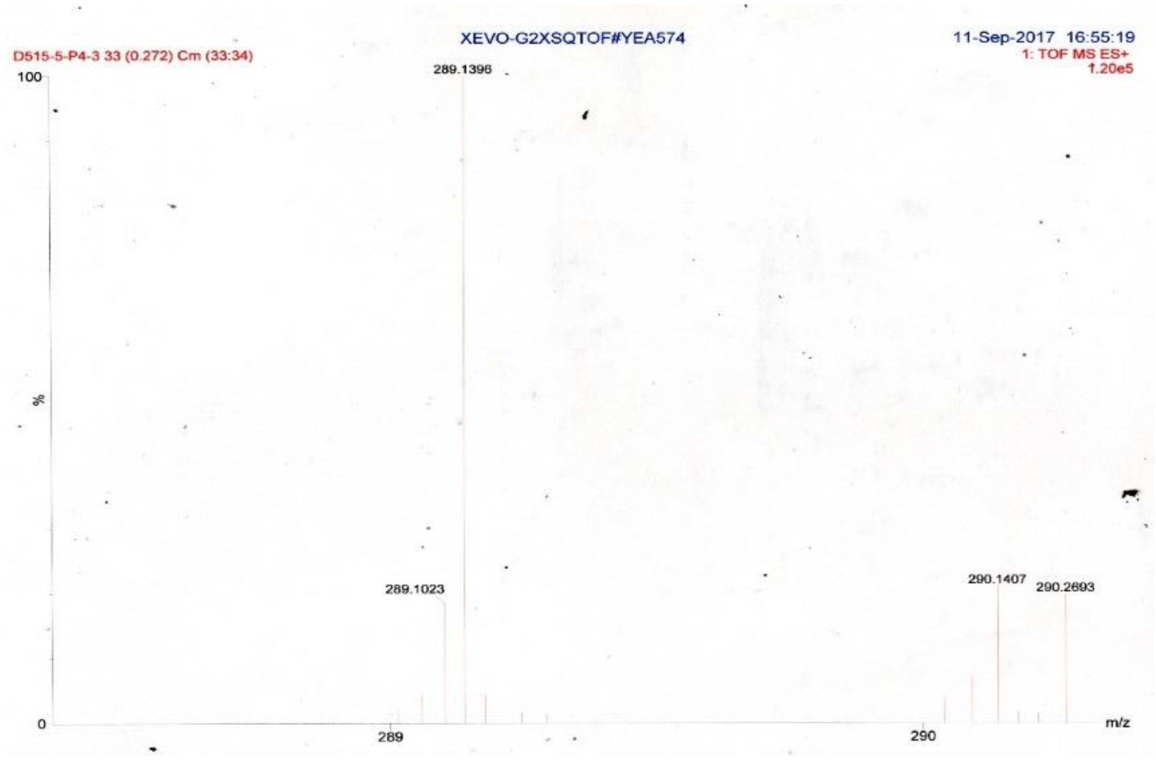

Figure S33. HRESIMS spectrum of compound **3**

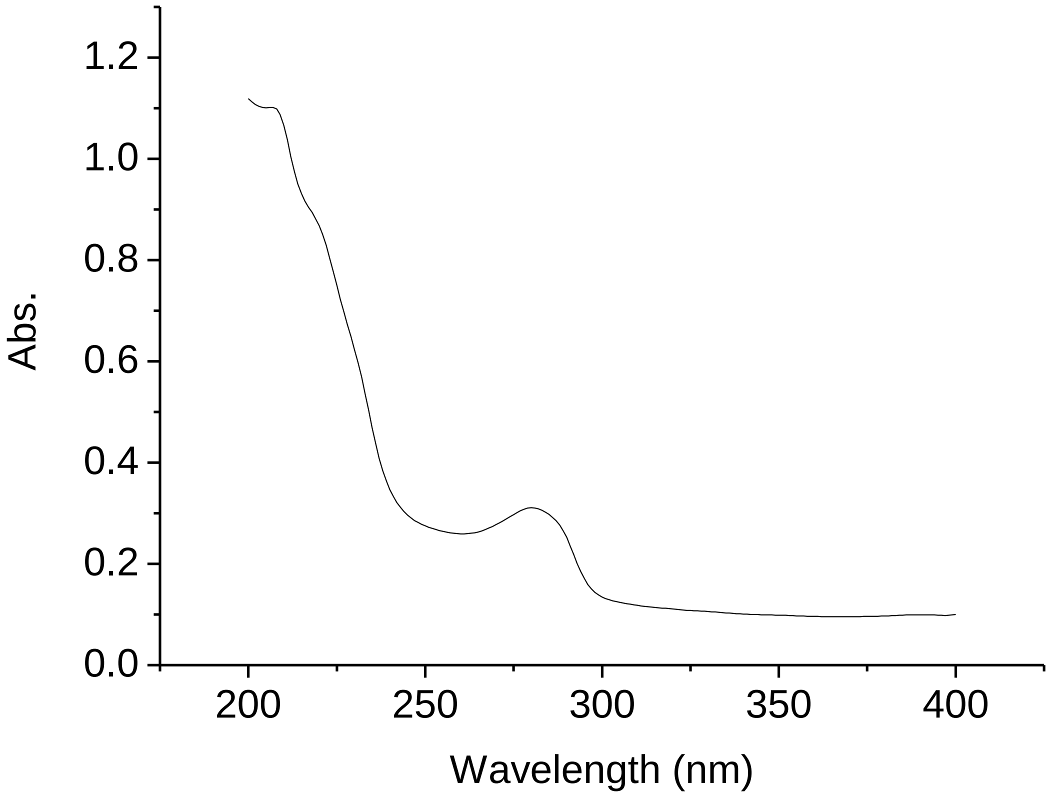

Figure S34. UV spectrum of compound **3**

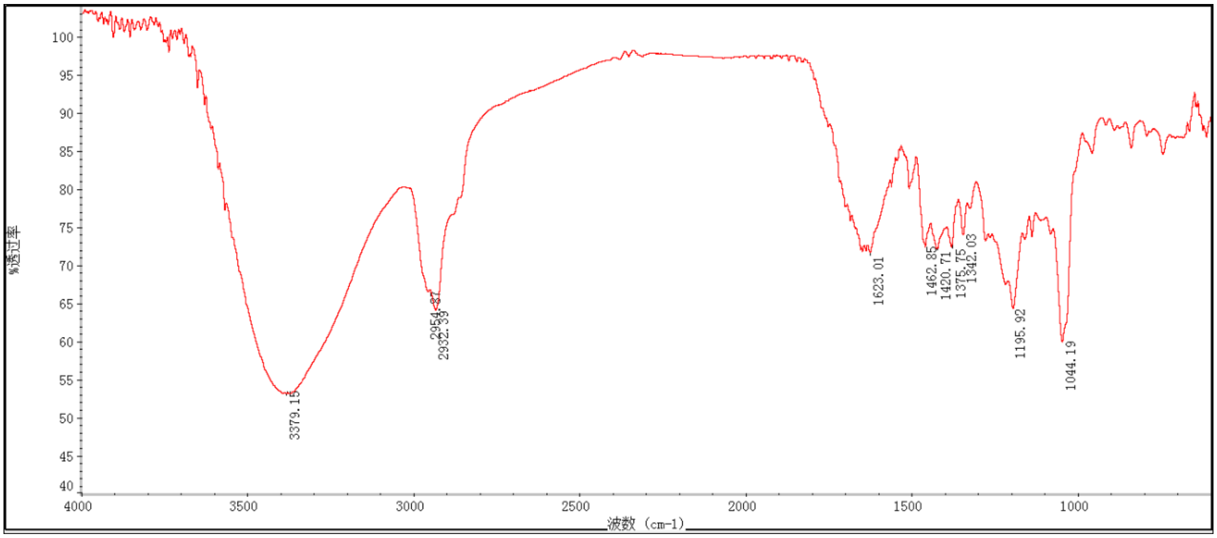

Figure S35. IR spectrum of compound **3**

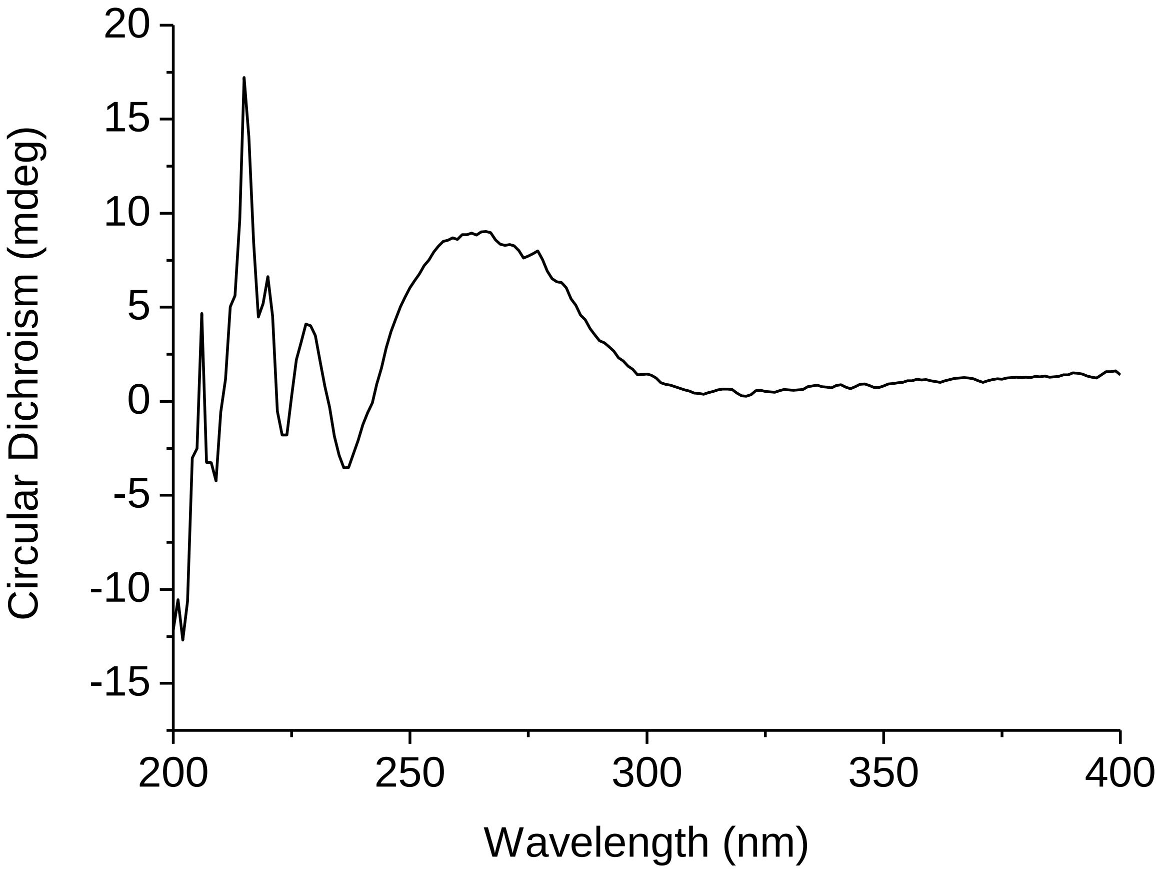

Figure S36. CD spectrum of compound **3**

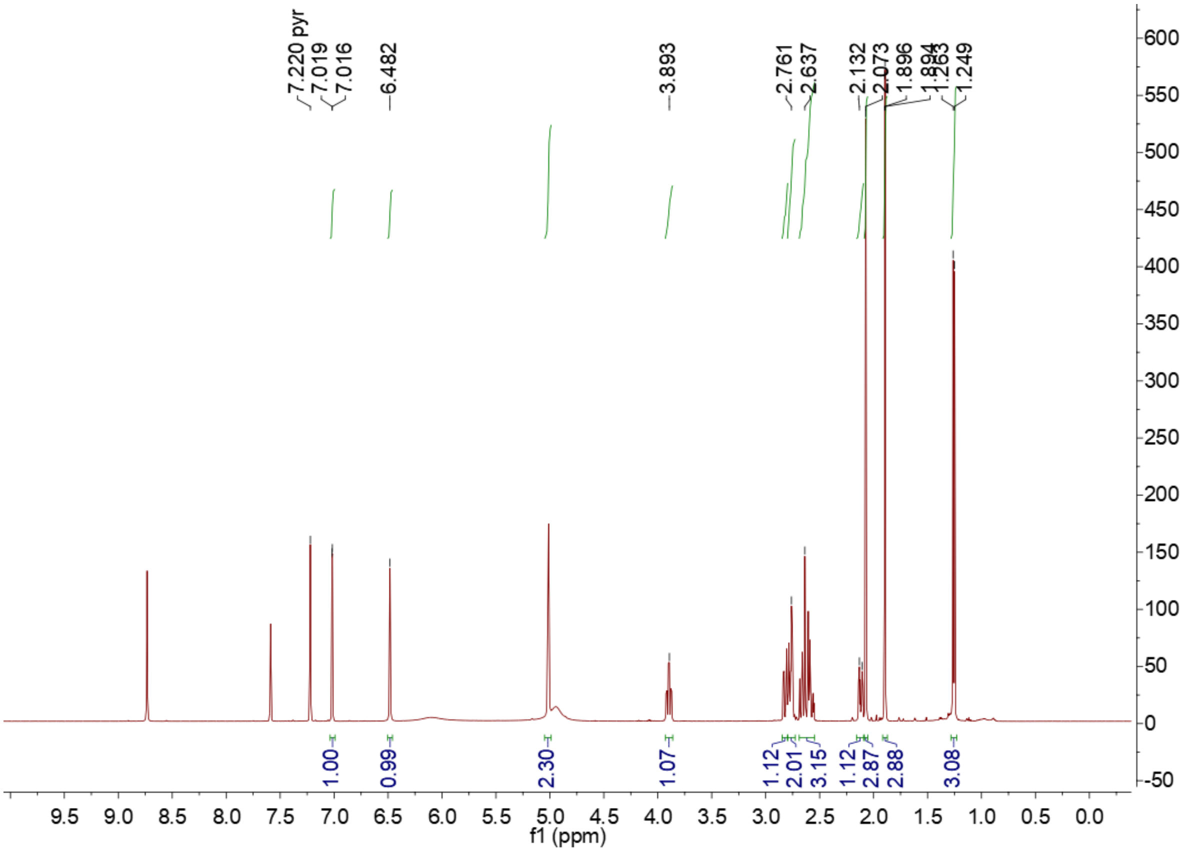

Figure S37. ^{1}H NMR spectrum of compound **4** (Pyridine-d_5)

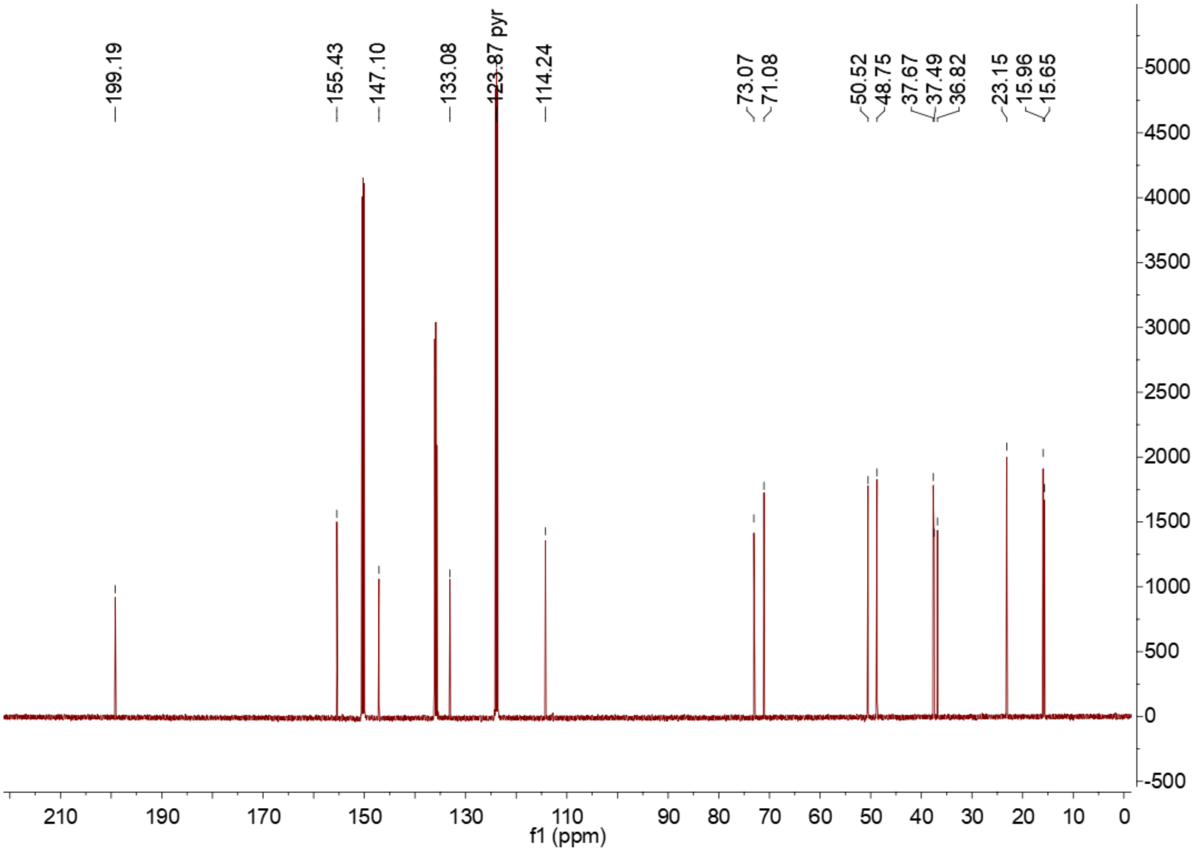

Figure S38. ^{13}C NMR spectrum of compound **4** (Pyridine-d_5)

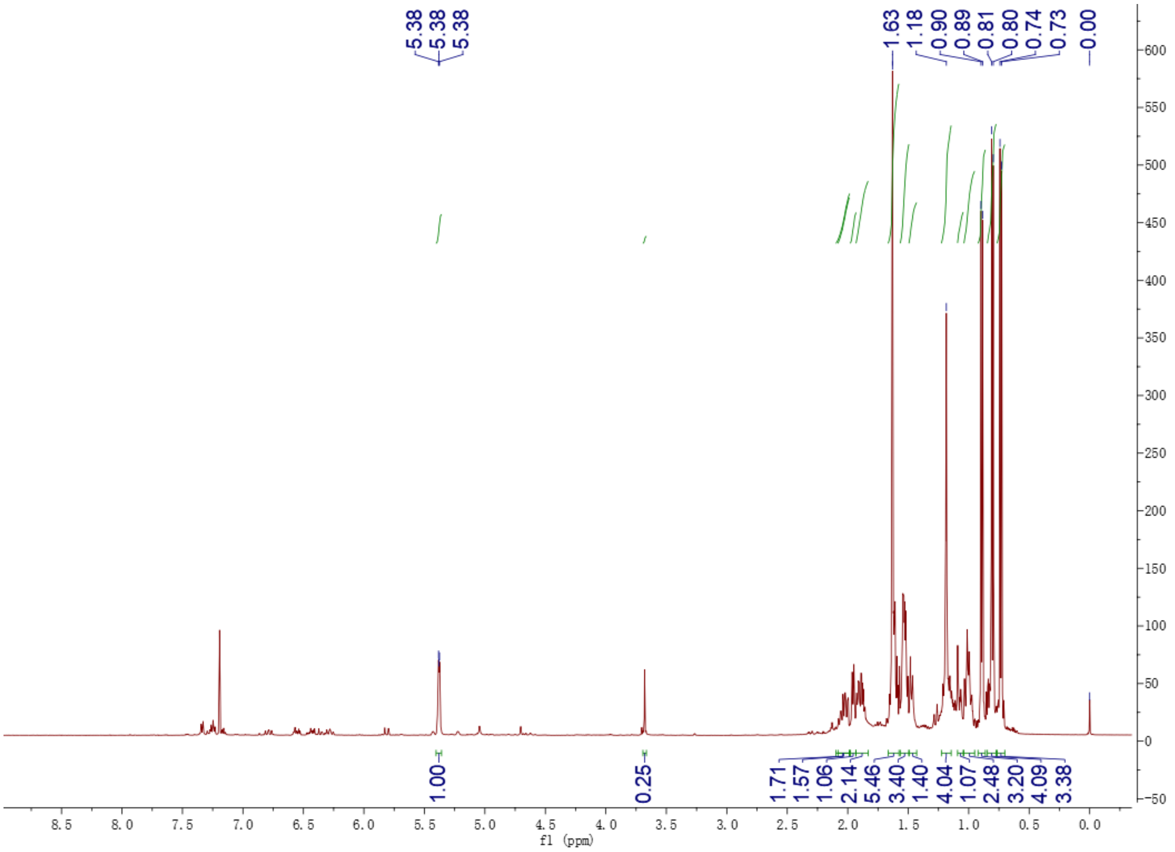

Figure S39. ^{1}H NMR spectrum of compound **5** (CDCL3)

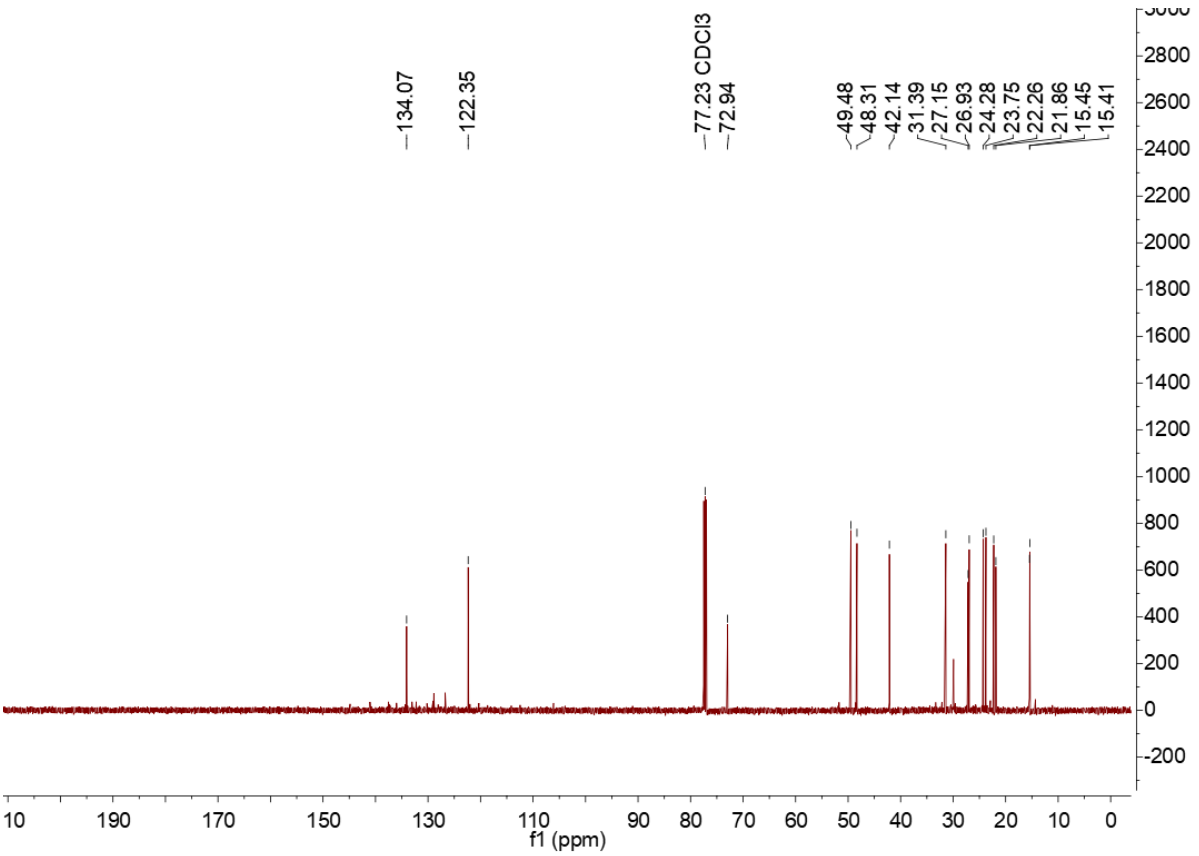

Figure S40. ^{13}C NMR spectrum of compound **5** ($CDCl_3$)

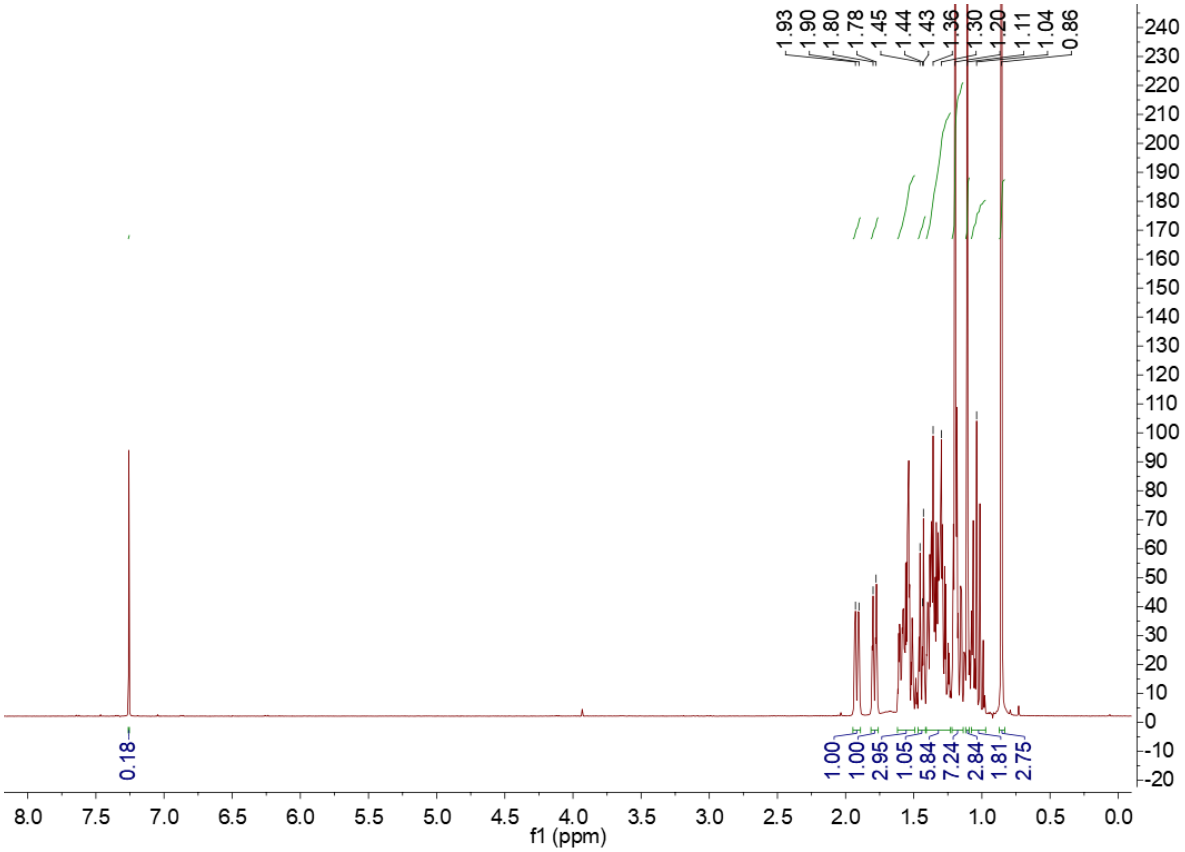

Figure S41. ^{1}H NMR spectrum of compound **6** ($CDCL_3$)

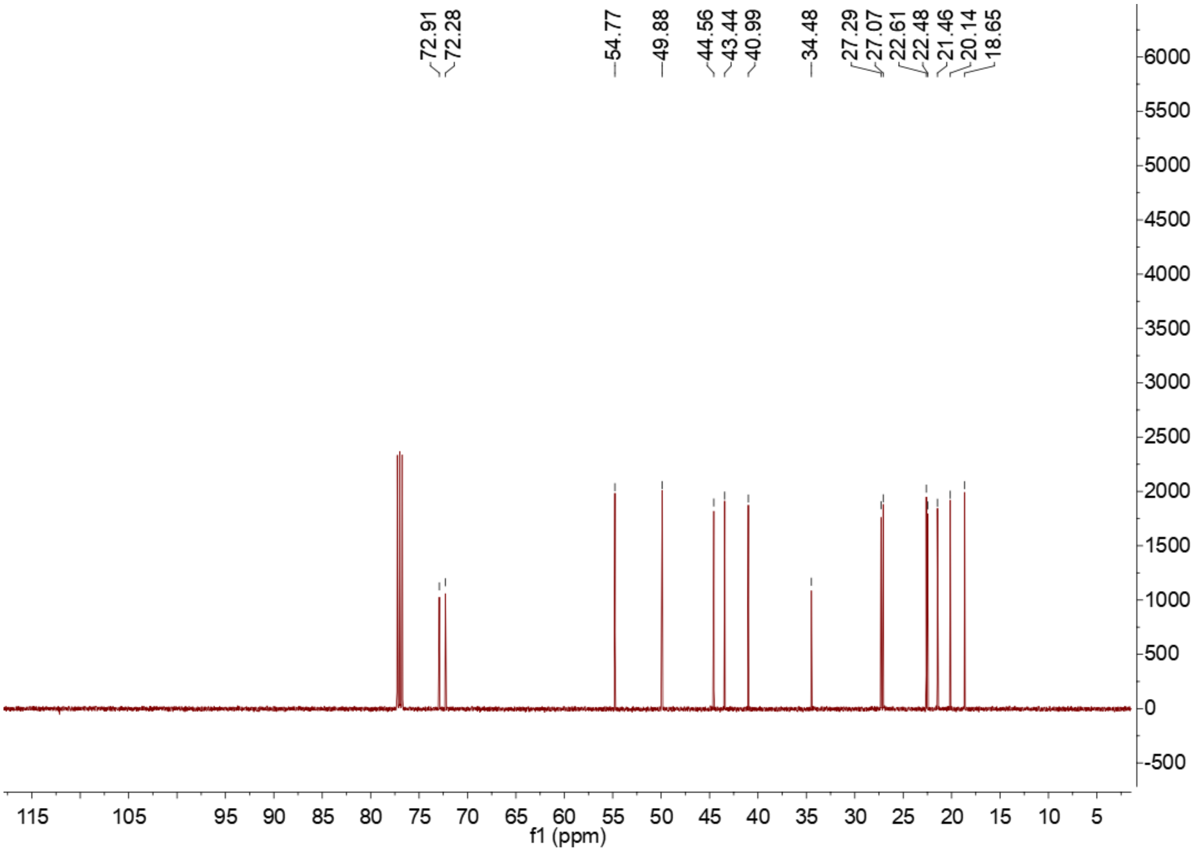

Figure S42. ^{13}C NMR spectrum of compound **6** ($CDCl_3$)

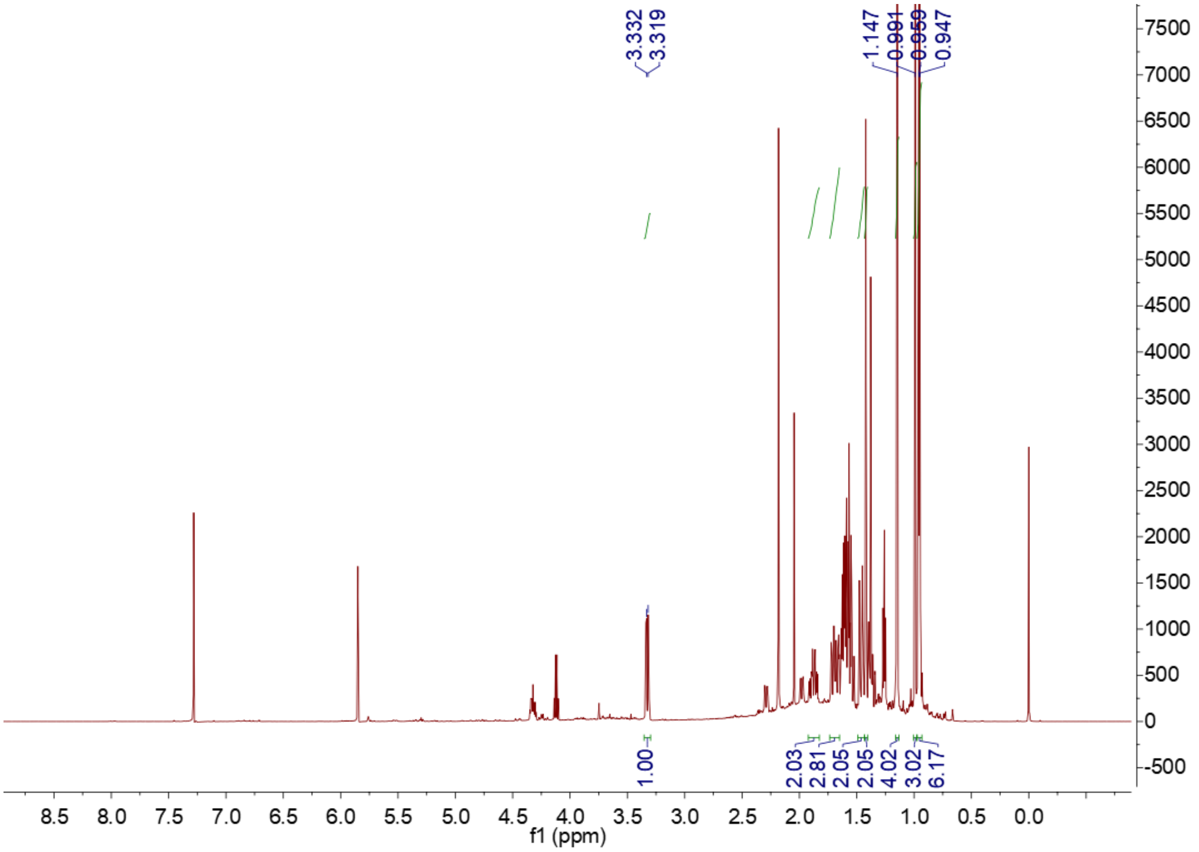

Figure S43. ^{1}H NMR spectrum of compound **7** (CDCL3)

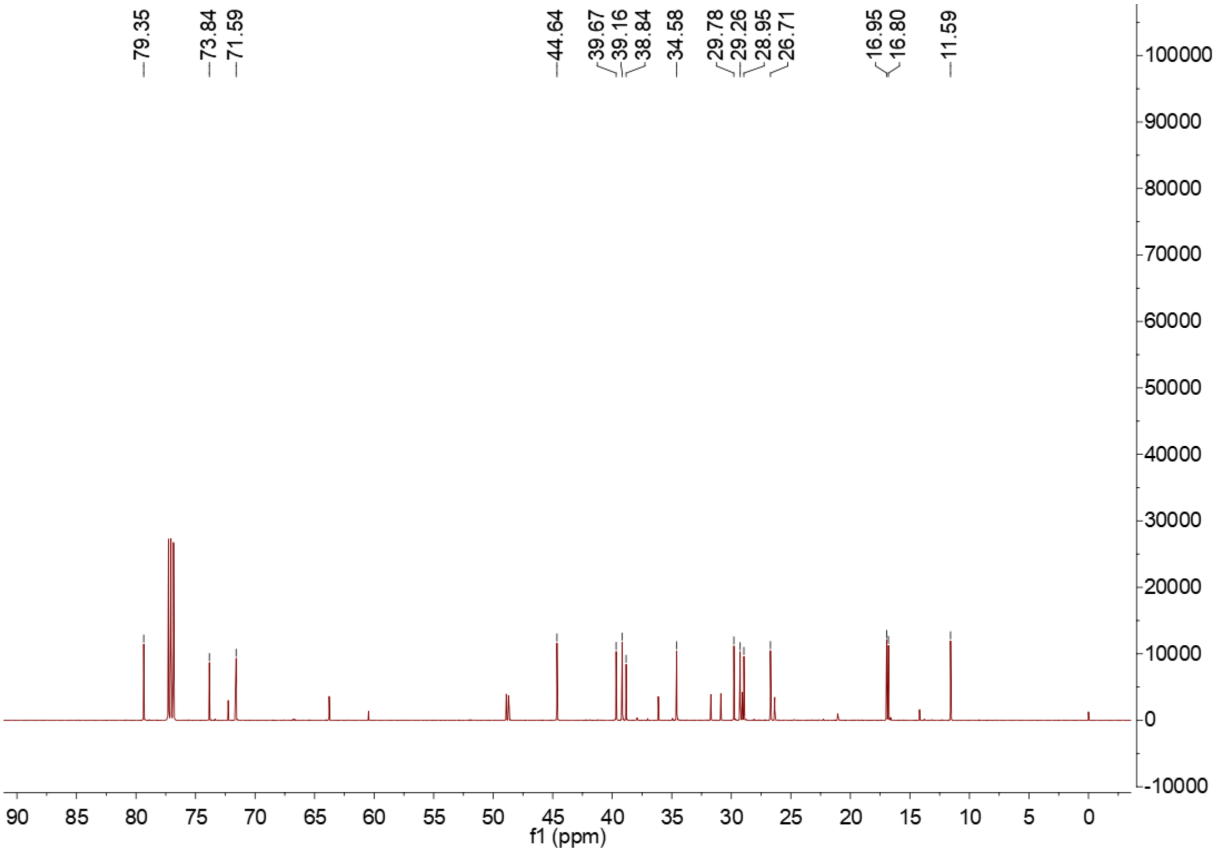

Figure S44. ^{13}C NMR spectrum of compound **7** ($CDCl_3$)

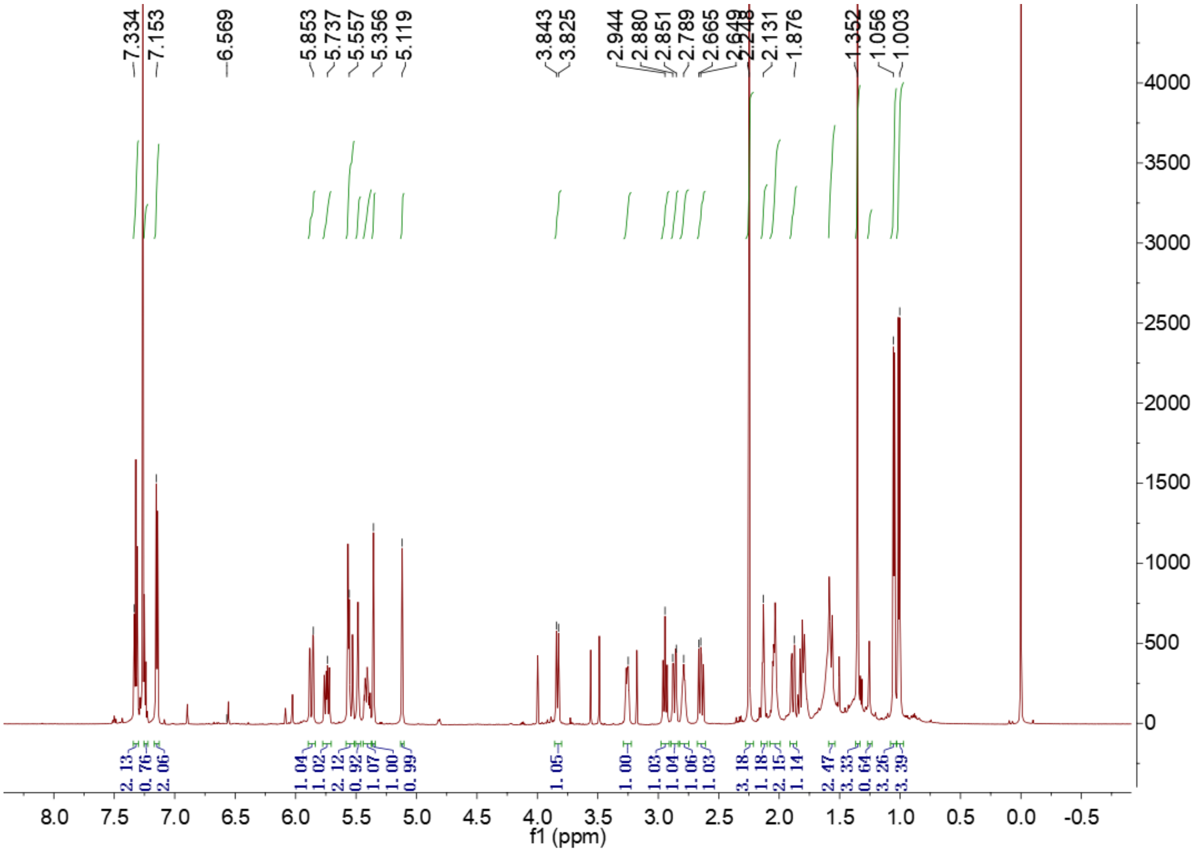

Figure S45. ^{1}H NMR spectrum of compound **8** ($CDCL_3$)

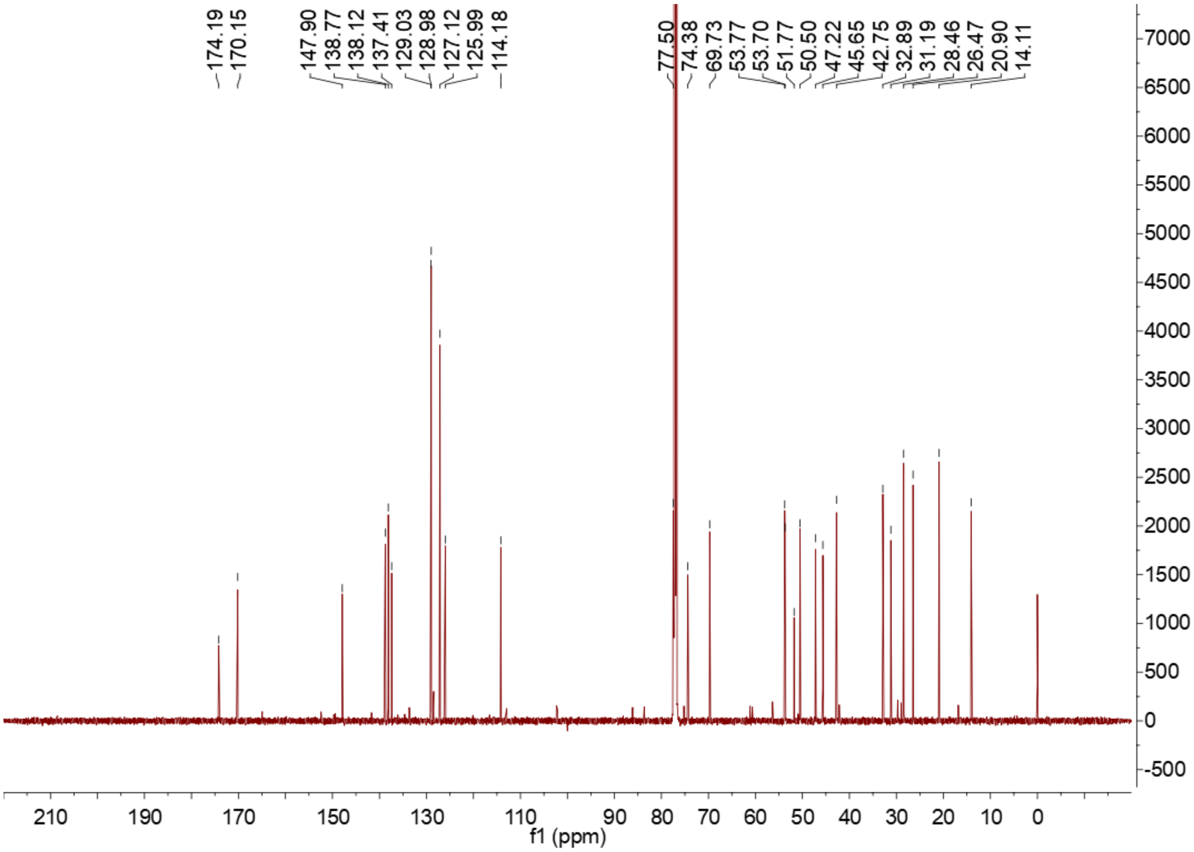

Figure S46. ^{13}C NMR spectrum of compound **8** ($CDCl_3$)
